# Advancing influenza virus treatment: *in vitro* and *ex vivo* studies of PI3K inhibitor-loaded lipid nanoparticles

**DOI:** 10.1016/j.mtbio.2025.102587

**Published:** 2025-11-26

**Authors:** Josefine Schroeder, Jana Ismail, Caroline T. Holick, Johannes Jungwirth, Laura Klement, Stephanie Hoeppener, Christian Kosan, Michaela Schmidtke, Bettina Löffler, Christine Weber, Ulrich S. Schubert, Carsten Hoffmann, Stephanie Schubert, Christina Ehrhardt

**Affiliations:** a Section of Experimental Virology, Institute of Medical Microbiology, Center for Molecular Biomedicine (CMB), Jena University Hospital, Jena, Germany; b Laboratory of Organic and Macromolecular Chemistry (IOMC), Friedrich Schiller University Jena, Jena, Germany; c Jena Center for Soft Matter (JCSM), Friedrich Schiller University Jena, Jena, Germany; d Institute of Molecular Cell Biology, Center for Molecular Biomedicine (CMB), Jena University Hospital, Friedrich Schiller University Jena, Jena, Germany; e Department of Biochemistry, Center for Molecular Biomedicine (CMB), Friedrich Schiller University Jena, Jena, Germany; f Institute of Medical Microbiology, Jena University Hospital, Jena, Germany; g Helmholtz Institute for Polymers in Energy Applications Jena (HIPOLE Jena), Jena, Germany; h Helmholtz-Zentrum Berlin für Materialien und Energie GmbH (HZB), Berlin, Germany

**Keywords:** Influenza A virus (IAV), Antivirals, Phosphatidylinositol 3-kinase (PI3K), Pictilisib, Lipid nanoparticle (LNP), poly(2-oxazoline) (POx), *Ex vivo*

## Abstract

Influenza A viruses (IAVs) remain a significant global health concern, causing seasonal outbreaks and pandemics with substantial morbidity and mortality, particularly among vulnerable groups. Annual vaccination serves as the primary preventative measure, but its limited effectiveness against emerging subtypes and insufficient coverage underscore the urgent need for novel antiviral strategies. Resistance to Food and Drug Administration (FDA)- and European Medicines Agency (EMA)-approved direct antivirals further emphasizes this necessity. Host-directed therapeutic strategies, such as those targeting phosphatidylinositol 3-kinases (PI3Ks), which are utilized by IAVs during replication, offer promising alternatives to reduce infection severity. In parallel, advances in nanotechnology have facilitated the use of lipid nanoparticles (LNPs) as efficient drug delivery systems, enhancing bioavailability, optimizing therapeutic stability, and enabling precise site-specific delivery to infected cells. This study investigates the combined use of pictilisib, a PI3K inhibitor, and LNPs, as a novel approach to combat IAV infections. Polyethylene glycol (PEG)- and poly(2-oxazoline) (POx)-Lipids were incorporated into the drug delivery carriers, with POx-Lipids emerging as promising alternatives due to allergenic concerns associated with PEG. Pictilisib was successfully encapsulated in LNPs and demonstrated comparable antiviral and anti-inflammatory properties to the free drug *in vitro*. Notably, *ex vivo* experiments revealed that POx-Lipid LNPs encapsulating pictilisib had a more potent effect on infection compared to free pictilisib, suggesting improved pharmacokinetics, drug stability, and targeted delivery. This integrated approach combining targeted PI3K inhibition and advanced nanocarrier technology represents a significant advance in addressing the challenges associated with IAV treatment, paving the way for further exploration of these strategies to combat infectious diseases.

## Graphical abstract

**Figure:**
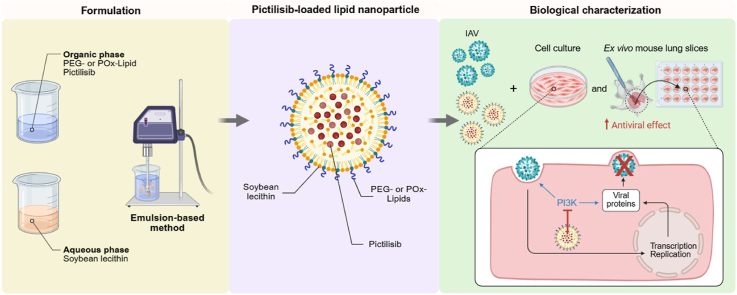

## 1 Introduction

Influenza viruses remain a persistent global health issue, causing seasonal outbreaks, pandemics, and zoonotic transmissions, with high morbidity and mortality rates, in particular among children, the elderly, and immunocompromised individuals [[1], [2], [3]]. According to the World Health Organization (WHO), influenza viruses are responsible for an estimated 1 billion cases annually, including 3 to 5 million severe cases and 290,000 to 650,000 deaths worldwide [4,5]. Influenza A viruses (IAVs), which belong to the family of the *Orthomyxoviridae*, are characterized by their single-stranded, negative-sense RNA genome enclosed within a lipid membrane envelope. These viral particles predominantly exhibit a spherical or filamentous shape with a size of 80–120 nm, although irregular morphologies may occasionally occur [6]. High mutation rates, due to the rapid genetic drift and shift of influenza viruses make control efforts challenging and significantly increase healthcare as well as economic burdens [7]. Annual influenza vaccination is the primary approach to manage seasonal outbreaks. However, challenges due to low vaccination coverage, inconsistent effectiveness, and the lack of protection against newly emerging subtypes persist [4,8]. This underscores an urgent need for the development of efficient antivirals to limit viral replication and reduce the burden of infection. Currently, three classes of antiviral drugs are approved for influenza virus treatment by the Food and Drug Administration (FDA) and European Medicines Agency (EMA): matrix 2 (M2) ion channel inhibitors, neuraminidase inhibitors (NAIs), and the cap-dependent endonuclease inhibitor baloxavir marboxil [9,10]. NAIs and baloxavir marboxil target both IAVs and influenza B viruses (IBVs), whereas M2 ion channel inhibitors are specific to IAVs [10]. While widespread resistance has emerged against M2 ion channel inhibitors, resistance development against drugs targeting viral proteins remains a concern, emphasizing the need for alternative therapies with higher resistance barriers [11].

Influenza viruses exploit host cell machinery for replication and activate intracellular signaling pathways to enhance dissemination [12]. Targeting these virus-supportive cellular factors has proven to be a promising strategy and offers the advantage of reducing the risk of resistance development through host-directed therapies [13]. One such key mediator is phosphatidylinositol 3-kinase (PI3K), together with its downstream effectors, protein kinase B (Akt) and mammalian target of rapamycin (mTOR), which play crucial roles in regulating cell proliferation, apoptosis, and cell metabolism and growth. Among the three PI3K classes (I (A and B), II, III), class I is of particular importance during cancer progression and viral infections [[14], [15], [16]]. In the context of cancer, PI3K signaling has been extensively studied and is known to promote tumor cell proliferation and contribute to resistance against chemotherapy, making it one of the most frequently dysregulated pathways and a central target in oncologic drug development [17]. For instance, fibroblast growth factor 2 (FGF2) has been shown to promote chemoresistance in colon cancer cells via activation of the PI3K/Akt pathway [18]. Beyond its role in cancer, PI3K activation has been documented in various viral infections, such as severe acute respiratory syndrome coronavirus 2 (SARS-CoV-2), where it activates several cytokines [19], as well as in human cytomegalovirus (HCMV) infections, where it initiates viral DNA replication [20]. Moreover, it has been observed in the regulation of vesicular uptake and trafficking of Ebola viruses [21], as well as in the human papillomavirus (HPV)-induced processes that contribute to tumor initiation and progression [22]. During influenza virus infection, PI3K plays several critical roles, including facilitating viral entry [23,24] and regulating endosomal acidification, which is essential for fusion between the viral and endosomal membranes [25]. Additionally, early activation is likely mediated by the viral RNA (vRNA) sensory pathway [26], while interactions of PI3K binding with the viral non-structural protein 1 (NS1) contribute to prevention of premature apoptosis [27]. It also supports viral ribonucleoprotein (vRNP) nuclear export, facilitating viral replication [28]. The broad spectrum of PI3K-dependent mechanisms in viral infections underscores its potential as a target for antiviral therapy.

Repurposing of approved drugs, as well as promising pharmaceutical substances currently being studied for other indications, has emerged as a promising strategy, as it can accelerate the research and development process, reduce costs, and minimize the risk of unintended side effects [29]. This approach has gained attention in recent years, in particular during the coronavirus disease 2019 (COVID-19) pandemic, where existing medications were evaluated for their potential antiviral effects [30]. We have recently shown that d,l-lysine-acetylsalicylate + glycine (LASAG), a nonsteroidal anti-inflammatory drug, exhibited antiviral efficacy against SARS-CoV-2 [31], while salts of procaine, a local anesthetic, demonstrated effectiveness against both SARS-CoV-2 and IAV infections *in vitro* [32,33]. Similarly, previous research has demonstrated the antiviral potential of pictilisib against IAVs [34,35]. Pictilisib, an investigational pan-class I PI3K inhibitor, also known as GDC-094, has been used in several phase I and II trials for combination therapy of advanced breast cancer and other solid tumors [[36], [37], [38]]. In a phase I study in patients with advanced solid tumors, pictilisib demonstrated antitumor activity, particularly in breast cancer models with phosphatidylinositol-4,5-bisphosphate 3-kinase, catalytic subunit α (PIK3CA) mutations, and/or human epidermal growth factor receptor 2 (HER2) amplification [36]. Additionally, it was shown to modulate the inflammatory response, presenting a promising candidate for the development of influenza treatment [34,35]. However, pictilisib and other PI3K inhibitors have been associated with significant toxicities, and symptoms such as skin rashes, nausea, fatigue, and diarrhea which has hindered their progression as a standalone therapeutic agent [[37], [38], [39], [40]]. Similarly, first generation PI3K inhibitors, such as wortmannin (a viridin derivative from soil bacteria) and LY294002 (a quercetin derivative), underwent basic research but failed to reach clinical trials due to suboptimal pharmacological properties [41,42]. Other challenges associated with small-molecule drugs are low water solubility and poor bioavailability, which hinder targeted delivery and result in undesired systemic distribution [38]. Thus, alternative approaches to drug development are urgently required, either by improving the pharmacological profile or by identifying more efficient routes of administration [41]. To overcome these limitations, the development of specialized nanocarrier systems is essential, as they can enhance drug solubility, improve bioavailability, and enable more effective and safer delivery.

Lipid nanoparticles (LNPs) have emerged as a versatile drug delivery platform due to their biocompatibility, ability to encapsulate hydrophobic and hydrophilic compounds, and potential for targeted delivery [[43], [44], [45]]. LNPs are being used for various indications, including cancer therapy, gene delivery, and vaccine development. Notably, LNPs play a crucial role in the success of mRNA-based COVID-19 vaccines, where they serve as carriers to protect and efficiently deliver mRNA into cells [[46], [47], [48]]. To enhance their stability, circulation time, and targeting capabilities, LNPs are being designed incorporating surface modifications, such as poly(ethylene glycol) (PEG) or alternative hydrophilic polymer coatings. These additions can create “stealth” LNPs that evade rapid clearance by the mononuclear phagocyte system, prolonging circulation time and improving drug biodistribution. These stealth properties are particularly valuable for antiviral therapies, as they enhance drug accumulation at infection sites while reducing systemic toxicity [[49], [50], [51], [52]]. However, recent findings demonstrated that PEGylated lipids such as 1,2-dimyristoyl-*rac*-glycero-3-methoxypolyethylene glycol-2000 (DMG-PEG2k) and α[2-(ditetradecylamino)-2-oxoethyl]-ω-methoxy-poly(oxy-1,2-ethanediyl) (ALC-0159) have been associated to hypersensitivity reactions including anaphylactic shocks [[53], [54], [55]]. As a result, poly(2-oxazoline)s (POx), in particular those with methyl and ethyl side chains, have gained a significant interest due to their similar properties as PEG in terms of biocompatibility, hydrophilicity and most importantly, the stealth effect [[56], [57], [58]]. For these reasons, POx-Lipids have been introduced as an alternative to PEG in LNPs. Various POx-Lipid formulations have been investigated in several studies, including POx analogues of ALC-0159, to assess the effects of different degrees of polymerization [59]. Additionally, novel heterotelechelic POx-Lipids, as well as POx-Lipids with monoacyl and diacyl terminal groups, have been explored [60,61]. Studying these modifications as well as comparing them in terms of safety and efficacy is of particular importance.

In our study, the aim was to successfully design a drug delivery carrier for the encapsulation of pictilisib to enhance its bioavailability and therapeutic efficacy. As nanocarrier-based delivery of PI3K inhibitors remains a largely unexplored area of research in the antiviral field, this work introduces a novel POx-Lipid-based nanocarrier as a biocompatible alternative to PEGylated particles, offering a new class of materials distinct but equally important to the existing vaccine lipids, that enables the encapsulation of the challenging-to-formulate PI3K inhibitor. To evaluate the antiviral potential of encapsulated pictilisib against IAVs, both *in vitro* and *ex vivo* studies were conducted. The findings suggest that encapsulating pictilisib holds significant promise as a therapeutic option for antiviral intervention. However, further testing in *in vivo* systems is required to explore its additional positive effects in greater detail.

## 2 Material and methods

### 2.1 Materials

Pictilisib (purity: 99.62 %) and 1,1′-dioctadecyl-3,3,3′,3′-tetramethylindocarbocyanine (DiI), a carbocyanine dye, were purchased from MedChemExpress (Sollentuna, Sweden). Stealth lipids were acquired from different sources: 1,2-dimyristoyl-*rac*-glycero-3-methoxypolyethylene glycol-2000 (DMG-PEG2k) was received from Avanti Polar Lipids (Alabaster, AL, USA) and α[2-(ditetradecylamino)-2-oxoethyl]-ω-methoxy-poly(oxy-1,2-ethanediyl) (ALC-0159) from Cayman Chemical (Ann Arbor, MI, USA). Poly(2-ethyl-2-oxazoline) (PEtOx_20_)-Lipid was synthesized in an optimized manner according to a published procedure [59]. Details are described in the Supplementary Information (SI; Scheme S1, Figs. S2 and S3). Soybean lecithin as a stabilizer has been procured from EMD-Millipore (Sigma-Aldrich, Darmstadt, Germany). Chloroform (CHCl_3_; anhydrous, >99 %) was purchased from Sigma-Aldrich (Darmstadt, Germany). Dimethyl sulfoxide (DMSO; 99.9 %) was acquired from Thermo Fisher Scientific (Schwerte, Germany). Labsolute syringe filters (13 mm, sterile, 0.45 μm, cellulose acetate (CA)) were purchased from Th. Geyer (Renningen, Germany). Milli-Q water was used in all stages of LNP preparation, purification, and characterization.

### 2.2 Formulation of LNPs

**Fig. 1:**
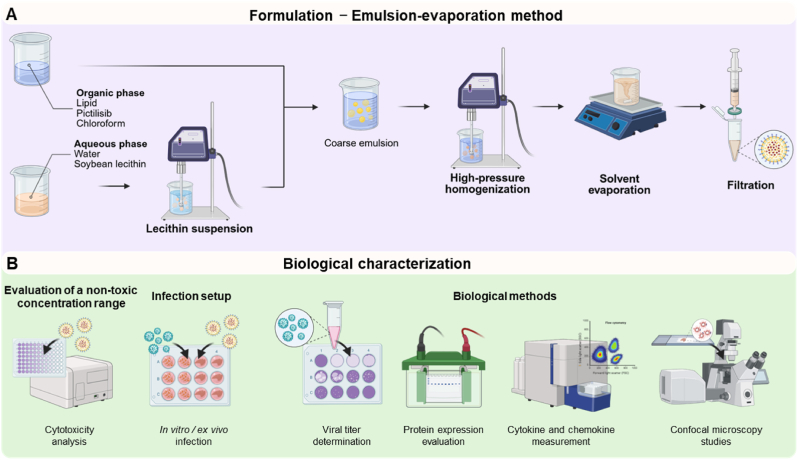
Scheme for (A) the formulation of the pictilisib-loaded LNPs using an emulsion-based technique and (B) the biological characterization methods.

LNPs were formulated using an oil-in-water single emulsion-evaporation technique, adapted and modified from Cheng et al. [62] (Fig. 1A).

To prepare pictilisib-loaded LNPs, pictilisib (0.833 mg, representing 1 % (w/w) of the total weight of lipid and stabilizer) and the stealth lipid (DMG-PEG2k, ALC-0159, or PEtOx_20_-Lipid, 7.5 mg) were dissolved in 0.5 mL chloroform. In parallel, as a stabilizer, soybean lecithin (75 mg) was resuspended in 5 mL Milli-Q water and ultrasonicated for 20 s to form a nanosuspension. Ultrasonication was performed using a probe sonicator (Hielscher Sonotrode S26d2, Ø 2 mm, length ∼120 mm) powered by an ultrasonic generator (UP200ST, Hielscher Ultrasonics, Teltow, Germany) at 100 % cycle, 100 W power and 20 % amplitude. Next, the organic phase, containing pictilisib and the selected stealth lipid, was added dropwise to the lecithin nanosuspension, followed by ultrasonication for 60 s. The sample was kept on ice throughout sonication to prevent overheating. The resulting emulsion was stirred at 800 rpm overnight under a fume hood to allow for complete solvent evaporation.

For DiI-loaded LNPs, the same formulation procedure was followed, except that DiI (0.25 % w/w of the total lipid and lecithin) was added instead of pictilisib. DiI was dissolved in the organic phase prior to emulsification.

All steps were carried out at room temperature (RT), and all samples were protected from light with aluminum foil throughout the preparation process. To remove any precipitated cargo and unencapsulated material, the LNP suspensions were purified by syringe filtration. First, 0.45 μm CA filters were pre-wetted with Milli-Q water to ensure optimal performance. The suspensions were subsequently passed through the filters using a syringe, applying gentle manual pressure to separate free material from the particles. The filtrates were collected for further characterization.

### 2.3 LNP characterization

Dynamic light scattering (DLS) and electrophoretic light scattering (ELS) were performed using a Zetasizer Ultra (Malvern Panalytical, Malvern, UK) to assess the particle size distribution and zeta potential (*ζ,* the electric charge on the surface) of the LNPs. Measurements were conducted with a 633 nm laser. Particle size and polydispersity index (PDI) were measured using polystyrene UV cuvettes (Brand, Wertheim, Germany), while zeta potential was determined using DTS1070 capillary cuvettes made of polycarbonate (Malvern Panalytical, Kassel, Germany). The analyses were carried out at a back-scattering angle of 174.7° and a temperature of 25 °C. The hydrodynamic diameter (d_h_) was derived from the intensity-weighted particle size distribution and reported as the z-average diameter. The particles were diluted (1:100) in Milli-Q water, with d_h_ and PDI measurements performed in five replicates, each consisting of 15 runs lasting 1.68 s. Zeta potential was determined from three measurements. A fluorescence filter was implemented when DiI-loaded particles were measured.

The capacity of the LNPs to encapsulate pictilisib was assessed using UV–Vis spectroscopy. Lyophilized samples were dissolved in DMSO in volumes matching the freeze-dried aliquots and left to stir for 15 min. Then, the samples were transferred to a Hellma Quartz 96-well plate (Hellma, Jena, Germany), and a standard curve of pictilisib was prepared. UV absorbance of pictilisib content was measured using the Infinite M200 Pro Plate Reader (Tecan Group, Männedorf, Switzerland) at 320 nm with a 3 × 3 multiple-read configuration and a 2000 μm well border.

To calculate the encapsulation efficiency (EE) and loading capacity (LC) of pictilisib-loaded LNPs, the following equations were employed:

Equation (1): Loading Capacity (LC%)

$$LC\;\left(\%\right)=\frac{mass\;of\;pictilisib\;recovered}{mass\;of\;total\;particles\;recovered}\times 100$$

Equation (2): Encapsulation Efficiency (EE%)

$$EE\;\left(\%\right)=\frac{LC\;calculated}{LC\;theoretical}\times 100$$

### 2.4 Cryogenic transmission electron microscopy (cryo-TEM)

Cryo-TEM images were acquired utilizing a Titan Krios G4 transmission electron microscope (Thermo Fisher Scientific, The Netherlands) at an acceleration voltage of 300 kV utilizing the 4k Ceta CMOS camera. Sample vitrification was conducted with a Vitrobot Mark IV. 9 μL of the particle solutions were applied onto precleaned (Pelco Easy Glow) Quantifoil grids (R2/2, Quantifoil, Jena, Germany). Liquid ethane was used as cryogen. After vitrification, the samples were mounted into autogrid carriers (Thermo Fisher Scientific, Waltham, MA, USA) and transferred to the microscope maintaining sample temperatures always below −175 °C. Images were acquired utilizing the Velox software (Thermo Fisher Scientific, The Netherlands).

### 2.5 Cell culture, viruses and substances

Madin-Darby canine kidney (MDCK) and the human lung adenocarcinoma cell line (Calu-3) were cultured in Minimum Essential Medium with Earle's salts and l-glutamine (EMEM; Anprotec, Bruckberg, Germany) supplemented with 10 % fetal calf serum (FCS; Anprotec, Bruckberg, Germany). Calu-3 cells were used as they closely resemble the human bronchial epithelium, making them a highly suitable *in vitro* model for studying viral infections. The IAV strains used in this study were A/Puerto Rico/8/1934 (A/H1N1 PR8), A/Jena/5258/2009 (A/H1N1pdm09 Jena5258) [63] and A/Wisconsin/67/2005 (A/H3N2 Wis67). MDCK cells were used to propagate the virus strains in Panserin 401 (PAN-Biotech, Aidenbach, Germany) supplemented with 0.2 μg mL^−1^ TPCK-trypsin (Sigma-Aldrich, Darmstadt, Germany). Pictilisib was dissolved in DMSO for biological experiments to a stock concentration of 1 mM.

### 2.6 Evaluation of drug-induced cytotoxicity using cell counting and LDH release assays

To determine the impact of free and LNP-encapsulated pictilisib on cytotoxicity, cell count and lactate-dehydrogenase (LDH) assays were conducted. These methods, along with additional biological techniques used in this study, are summarized in Fig. 1B. For the cell count, Calu-3 cells (500,000 cells/well) were seeded in 24-well plates, cultured for 24 h, and afterwards treated with the indicated concentrations or respective volumes of pictilisib, non- and pictilisib-loaded LNPs as well as the pictilisib-solvent control (DMSO) for 24 h at 37 °C with 5 % CO_2_. The number of viable cells was subsequently quantified using the Countess II (Invitrogen, Dreieich, Germany). For this, a 1:1 ratio of cell suspension and 0.1 % trypan blue (Sigma-Aldrich, Darmstadt, Germany) solution was used. Cells treated solely with EMEM supplemented with 10 % FCS served as a reference and were set to 100 %. LDH release was analyzed using the CyQUANT LDH Cytotoxicity Assay kit (Thermo Fisher Scientific, Schwerte, Germany). For this purpose, Calu-3 cells (50,000 cells/well) were seeded into 96-well plates 24 h prior to use. Afterwards, cells were treated with the indicated concentrations or respective volumes of pictilisib, non- and pictilisib-loaded LNPs as well as the pictilisib-solvent control (DMSO) in 100 μL EMEM supplemented with 10 % FCS and incubated at 37 °C with 5 % CO_2_. As a control for maximum LDH release, 10 μL of lysis buffer provided by the kit were added after 24 h of compound-untreated growth, and cells were incubated for 45 min at 37 °C, 5 % CO_2_. Subsequently, LDH release from the supernatants was quantified according to the manufacturer's protocol. LDH release from compound-treated cells was compared to maximum LDH release from control cells treated with lysis buffer, which was set to 100 %. Samples exclusively treated with EMEM supplemented with 10 % FCS were set to 0 %. Absorbance was measured at 490 nm and 680 nm using a FLUOstar Omega plate reader (BMG Labtech, Ortenberg, Germany) with the corresponding Omega series software v5.50 R4. Background absorbance at 680 nm was subtracted from that at 490 nm.

### 2.7 Viral infection *in vitro*

For viral infection *in vitro*, Calu-3 cells were seeded in 12-well plates (500,000 cells/well) and cultivated for 36 h at 37 °C, 5 % CO_2_. Cells were washed with phosphate-buffered saline (PBS; Carl Roth, Karlsruhe, Germany) and subsequently pretreated with the indicated concentrations or respective volumes of compounds or solvent controls for 1 h. After that, medium was removed, and the cells were either left uninfected or infected with the indicated multiplicity of infection (MOI) of the indicated IAV strain in 250 μL PBS/BA (PBS supplemented with 0.2 % bovine serum albumin (BSA; Carl Roth, Karlsruhe, Germany), 1 mM MgCl_2_ (Sigma-Aldrich, Darmstadt, Germany) and 0.9 mM CaCl_2_ (Sigma-Aldrich, Darmstadt, Germany)) for 30 min at 37 °C and 5 % CO_2_. Then, cells were washed with PBS and incubated with the indicated concentrations or respective volumes of compounds and solvent controls in EMEM/BA (EMEM supplemented with 0.2 % BSA, 1 mM MgCl_2_, 0.9 mM CaCl_2_) with 0.2 μg mL^−1^ TPCK-trypsin until the indicated time points post infection (p.i.).

### 2.8 Ex vivo mouse lung model

For the *ex vivo* mouse lung model, female 10-week-old wild-type C57BL/6JRj mice (Janvier Labs, Le Genest-Saint-Isle, France) were used with the approval of the Thuringian State Office for Consumer Protection as part of a euthanasia notice (twz09-2024). The mice were euthanized by injecting an overdose of ketamine and xylazine. After opening the chest cavity and exposing the lung, the blood was drained through the heart, and the lung was flushed via the trachea with Hanks’ Balanced Salt Solution (HBSS; Capricorn Scientific, Ebsdorfergrund, Germany). Following that, 2 % low-melt agarose (Carl Roth, Karlsruhe, Germany) was intratracheally injected into the lungs. After the agarose thickened, lungs were extracted and stored in HBSS supplemented with 1 % penicillin/streptomycin (pen/strep; 100 U mL^−1^/0.1 mg mL^−1^, Sigma-Aldrich, Darmstadt, Germany) at 4 °C. Using a vibratome (Leica VT1200S, Leica, IL, USA), lung slices with a thickness of 200 μm were prepared, transferred to 48-well plates, and cultured in DMEM/F-12 (Anprotec, Bruckberg, Germany) supplemented with 1 % pen/strep and 10 % FCS at 37 °C and 5 % CO_2_. For LNP uptake studies, lung slices were treated as described in Section 2.12. For infection experiments, the medium was changed after 24 h and lung slices were infected with the A/H1N1pdm09 Jena5258 strain, which was adjusted to 2,000,000 plaque forming units (PFU) mL^−1^ in 250 μL DMEM/F-12/BA (DMEM/-F12 supplemented with 0.2 % BSA, 1 mM MgCl_2_, 0.9 mM CaCl_2_), supplemented with 1 % pen/strep. After an infection period of 3 h at 37 °C and 5 % CO_2_, the slices were washed with PBS and cultured in DMEM/F-12/BA supplemented with 1 % pen/strep and 0.2 μg mL^−1^ TPCK-trypsin for 10, 24, 48 or 72 h without treatment or only for 48 h with treatment of 0.5 μM pictilisib, POx-Lipid (pictilisib) or the respective volumes of controls (DMSO, POx-Lipid).

### 2.9 Viral titer quantification by plaque assay

Viral titers were determined by plaque assay. MDCK cells (2,000,000 cells/6-well for *in vitro* (Section 2.7) or 1,000,000 cells/12-well for *ex vivo* experiments (Section 2.8)) were seeded 24 h before infection to achieve a confluent layer. Cells were washed with PBS and subsequently infected with serial dilutions of the sample prepared in PBS/BA supplemented with 1 % pen/strep for 30 min at 37 °C and 5 % CO_2_. Following incubation, the virus solution was removed and replaced with prewarmed soft agar containing MEM (Gibco, Waltham, MA, USA) supplemented with 0.2 % BSA, 0.01 % DEAE dextran (Pharmacia Biotech, Freiburg im Breisgau, Germany), 0.2 % NaHCO_3_ (Gibco, Waltham, MA, USA), 1 % pen/strep, 0.2 μg mL^−1^ TPCK trypsin and 0.9 % agar (Oxoid, Wesel, Germany). The plates were incubated at 37 °C and 5 % CO_2_ for three days. PFU were visualized and counted using neutral red (Sigma-Aldrich, Darmstadt, Germany) dissolved in PBS.

### 2.10 Detection of protein expression by Western blot analysis

Western blot analysis was performed to analyze protein expression. For this purpose, cells were lysed with triton lysis buffer (20 mM Tris-HCl (pH 7.4), 137 mM NaCl, 10 % glycerol, 1 % Triton-X-100, 2 mM EDTA, 50 mM β-glycerophosphate, 20 mM sodium pyrophosphate) containing protease inhibitors (0.2 mM Pefabloc (Sigma-Aldrich, Darmstadt, Germany), 5 μg mL^−1^ aprotinin (Carl Roth, Karlsruhe, Germany), 5 μg mL^−1^ leupeptin (Sigma-Aldrich, Darmstadt, Germany), 1 mM sodium vanadate (Sigma-Aldrich, Darmstadt, Germany) and 5 mM benzamidine (Sigma-Aldrich, Darmstadt, Germany)) for 30 min at 4 °C. Cell lysates were transferred to microcentrifuge tubes and centrifuged at 14,000 rpm for 10 min at 4 °C. Supernatants were used to determine protein content using Protein Assay Dye Reagent Concentrate (BioRad, Berkeley, CA, USA). Protein concentrations were equally adjusted, lysates were supplemented with 5× Laemmli buffer and incubated for 10 min at 95 °C. Lysates and protein marker (PageRuler Prestained Protein Ladder; Thermo Fisher Scientific, Schwerte, Germany) were loaded on a 10 % SDS-PAGE and subsequently blotted onto a 0.2 μm nitrocellulose membrane (Amersham Protran, Marlborough, MA, USA). The membranes were then blocked with 2 % milk for 1 h. Proteins were detected using the primary antibodies (1:1000) listed in Table 1. WesternSure goat anti-rabbit HRP, WesternSure goat anti-mouse HRP or IRDye 680RD goat anti-mouse IgG (LI-COR Bioscience, Bad Homburg, Germany) were used as secondary antibodies at a dilution of 1:5000. Membranes were incubated in Pierce ECL Western Blotting Substrate (Thermo Fisher Scientific, Schwerte, Germany) and processed with the FusionFX6.Edge (Vilber Lourmat, Eberhardzell, Germany). Protein levels were quantified and normalized to the vinculin loading control using Fiji ImageJ 1.54i [64].

**Table 1:** Primary antibodies used for Western blot analysis.

Target	Manufacturer
pAkt (S473)	Cell signaling Technology (9271)
vinculin	Santa Cruz (73614)
pGSK-3β (Ser9)	Cell signaling Technology (5558)

### 2.11 Fluorescence plate reader analysis for LNP uptake studies

For cell-free LNP studies, DiI-loaded LNPs were diluted in EMEM/BA for serial dilutions of 0.1 mg mL^−1^, and subsequently diluted in a 1:1 ratio in 96-well black glass-bottom plates (Greiner Bio-One, Kremsmuenster, Austria). For cell-based LNP uptake studies, Calu-3 cells (50,000 cells/well) were seeded in EMEM supplemented with 10 % FCS in 96-well black glass-bottom plates 36 h prior to the experiment. Cells were washed with PBS and subsequently 0.1 mg mL^−1^ DiI-loaded LNPs in EMEM/BA or only EMEM/BA were added for 1 h up to 24 h and incubated at 37 °C and 5 % CO_2_. Cells were washed twice with PBS to remove the non-internalized LNPs. The fluorescence (excitation 540 nm, emission 610 nm, gain 130, bottom reading) was measured using a TECAN Infinite 200 (Tecan, Crailsheim, Germany) plate reader.

### 2.12 Histological analysis by confocal (immuno)fluorescence microscopy

For LNP uptake studies, Calu-3 cells (200,000 cells/well) were seeded or mouse lung slices were transferred into chambered 8-well cover slips (ibidi GmbH, Gräfelfing, Germany) in culture media. Cells were incubated for 24 h at 37 °C and 5 % CO_2_. The Calu-3 cells or *ex vivo* mouse lung slices were subsequently incubated with only medium or 0.1 mg mL^−1^ DiI-loaded LNPs in EMEM/BA or DMEM/F-12/BA supplemented with 1 % pen/strep (for the mouse lung slices) for the indicated time points. After that, the samples were washed three times. This process ensures that only the internalized particles are detected and avoids interference from non-internalized LNPs. For staining of the nuclei, samples were incubated in EMEM/BA or DMEM/F-12/BA supplemented with 1 % pen/strep containing 5 μg mL^−1^ BisBenzimide H 33342 trihydrochloride (Hoechst; 1:5000, Merck, Darmstadt, Germany) for 20 min.

For *ex vivo* mouse lung infection immunofluorescence studies, *ex vivo* mouse lung slices were cultivated in 48-well plates and infected as described in Section 2.8. At 48 h p.i., the slices were fixed for 1 h in 3.7 % formaldehyde (FA; Sigma-Aldrich, Darmstadt, Germany) at RT, permeabilized with 0.1 % Triton-X-100 for 1 h at RT and blocked with 10 % normal goat serum (NGS; Sigma-Aldrich, Darmstadt, Germany) for 1 h. Subsequently, the primary antibodies mouse anti-IAV nucleoprotein (NP; MCA400; BioRad, Berkeley, CA, USA), rabbit anti-IAV M2 (GTX125951; GeneTex, Downers Grove, IL, USA) and anti-rat CD68 (14-0681-82; Thermo Fisher Scientific, Schwerte, Germany) were added at a ratio of 1:200 for 1 h at RT. Alexa Fluor 488-conjugated goat anti-mouse IgG polyclonal antibody (115-545-146; Dianova, Hamburg, Germany), Alexa Fluor 488-conjugated goat anti-rabbit IgG polyclonal antibody (111-545-144; Dianova, Hamburg, Germany) and Cyanine3 (Cy3) goat anti-rat IgG cross-adsorbed secondary antibody (A10522; Thermo Fisher Scientific, Schwerte, Germany) were added as secondary antibodies for 1 h at RT in the dark. Finally, lung slices were mounted with fluorescence mounting medium (Agilent, Santa Clara, CA, USA) on glass microscope slides.

Imaging of either Calu-3 cells or *ex vivo* mouse lung slices for LNP uptake studies or infected and FA-fixed *ex vivo* mouse lung slices was performed using an inverted laser scanning confocal microscope (DMi8 TCS SP8, Leica microsystems, Wetzlar, Germany) operated with the Leica Application Suite X (version 3.5.5.19976). Images were acquired with a 63x oil immersion objective, with zoom factor 3 for images of the cells and the 10x objective for images of lung slices, line average 3, and 400 Hz in 2048 × 2048 pixel format. The Hoechst and DiI channels were excited sequentially at 405 nm and 561 nm, respectively, and emission was collected in the spectral intervals of 566 nm–650 nm and 410 nm–470 nm with hybrid photo (HyD) detector. Brightfield images were collected with a separate photomultiplier (PMT) detector. To capture IAV NP or M2 and CD68, channels were sequentially excited at 488 nm, 633 nm and 561 nm, respectively, and emission was collected in the spectral intervals of 500 nm–550 nm with HyD detector, 640 nm–674 nm with HyD detector, 570 nm–620 nm with PMT detector, 640 nm–656 nm with HyD detector. Images were later processed with Fiji ImageJ 1.54i [64] to adjust and set identical brightness values over the entire collection of images for all samples and time points within one experiment.

### 2.13 Quantification of immune response via flow cytometry

Flow cytometry analysis was performed using a LEGENDplex Human Anti-Virus Response Panel (740349; BioLegend, Amsterdam, The Netherlands) assay according to the manufacturer's protocol. 25 μL of the supernatant of the in Section 2.7 described experiments were incubated with premixed beads overnight at RT on a plate shaker. The detection antibodies were incubated for 1 h, followed by incubation with streptavidin, R-Phycoerythrin-conjugate (SAPE), for 30 min at RT on a plate shaker. Measurements were carried out on a BD FACSLyri flow cytometer (BD Bioscience, Heidelberg, Germany). The data analysis was performed using the LEGENDplex webtool powered by QOGNIT (San Carlos, CA, USA) (https://legendplex.qognit.com/).

### 2.14 Statistical analysis

Statistical analysis was performed using Prism v9.3.1 (GraphPad Software, San Diego, CA, USA). Statistical methods are described in the figure legends.

## 3 Results and discussion

### 3.1 Formulation and characterization of pictilisib-loaded LNPs

Given pictilisib's unfavorable physicochemical properties, there is a need for an effective nanocarrier system to improve its delivery and to ensure its therapeutic benefit. However, encapsulating pictilisib has proven to be challenging, as multiple attempts with polymeric nanocarriers (poly(d,l-lactic-*co*-glycolic acid) (PLGA), PEG-PLGA, poly(2-ethyl-2-oxazoline)-*b*-poly(l-lactide) (PEtOx-*b*-PLA [65]), acetalated dextran (AcDex)) and hybrid lipid-polymer nanocarriers (PLGA and (1,2-distearoyl-sn-glycero-3-phosphoethanolamine) (DSPE)-PEG) have failed, as shown in Table S1 (more details about the materials in the SI). In these formulations, pictilisib tended to precipitate, likely due to its low log P value in octanol-water (1.96) [66], preventing effective entrapment in hydrophobic polymer structures (Fig. S1).

To address this problem and to design a suitable nanocarrier, we sought to identify materials that can cater to the specific characteristics of pictilisib to optimize its encapsulation. Based on its molecular structure, pictilisib exhibits amphiphilic characteristics, with the sulfonamide moiety contributing to its hydrophilicity. In addition, pictilisib comprises at least five nitrogen atoms that are prone to protonation in water. As such, starting with and adapting the protocol by Cheng et al. [62], a suitable lipid-based nanocarrier was designed for pictilisib encapsulation. Soybean lecithin was selected as a stabilizer due to its amphiphilic nature and its key roles in structural integrity, steric stabilization, and biocompatibility as a naturally occurring phospholipid [[67], [68], [69], [70]]. Additionally, different stealth lipids were tested, starting with DMG-PEG2k, a widely used lipid for genetic material encapsulation [71,72]. Notably, this PEGylated lipid is employed in the LNP formulation of the mRNA-based vaccine Spikevax (Moderna), but it is not yet FDA- or EMA-approved for small molecules or protein drugs [73]. ALC-0159, another PEGylated lipid used in Comirnaty (BioNTech/Pfizer) vaccines, has been tested as well. While these two lipids are similar in their function of providing a hydrophilic PEG moiety, they differ in their lipid anchors and structural properties. DMG-PEG2k has a dimyristoyl glycerol backbone with ester bonds, leading to faster desorption from particles, favoring faster uptake and endosomal escape, while ALC-0159 has a more stable ditetradecylacetamide structure with amide bonds, promoting longer retention and enhanced nanoparticle stability [74,75]. The use of these lipids containing PEG as a stealth moiety offers the advantage of preventing opsonization of the nanocarriers and reducing their clearance by the reticuloendothelial system, thereby extending their blood circulation time [76]. Given the immunogenicity of PEG in humans and the development of anti-PEG antibodies, as reported by a growing body of literature [[77], [78], [79]], there is a need for alternative polymers, such as polyoxazoline (POx), particularly poly(2-ethyl-2-oxazoline) (PEtOx), which shows promise as a PEG substitute [80,81]. Therefore, PEtOx_20_-Lipid, a POx analogue of ALC-0159, with a molar mass of M_n_ = 2000 g mol^−1^, was synthesized and added to the study as a potential alternative for the PEGylated lipids. It was synthesized using a modified procedure based on a previously published method [59]. The polymers were characterized by means of proton nuclear magnetic resonance (^1^H NMR), size exclusion chromatography (SEC) and matrix-assisted laser desorption/ionization time-of-flight mass spectrometry (MALDI-TOF MS), with the corresponding data for the precursors as well as the lipid available in the SI (Figs. S2 and S3).

**Fig. 2:**
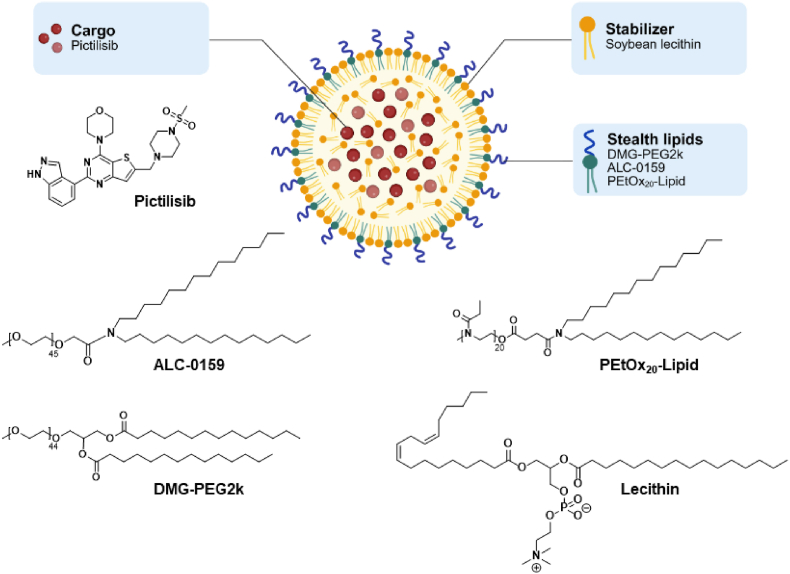
Schematic representation of the proposed structure of the LNPs as well as the chemical structures of pictilisib, lecithin, and the investigated PEG- and POx-Lipids.

Given all the materials mentioned, LNPs, which consist of a lipid-based matrix capable of accommodating both hydrophilic and hydrophobic drugs, were proposed as a suitable delivery system for pictilisib encapsulation, which we report in this study (Fig. 2). The three different stealth lipids were tested in separate formulations. For ease of reporting, DMG-PEG2k-based LNPs will be referred to as PEG-Lipid 1, ALC-0159 as PEG-Lipid 2, and PEtOx_20_-Lipid as POx-Lipid in the following discussion.

The properties of the formulated LNPs with encapsulated pictilisib and non-loaded particles as reference are described in Table 2 as reported by dynamic light scattering (DLS) and electrophoretic light scattering (ELS) measurements. Particle size was consistent throughout different batches and ranged between approximately 200 nm and 250 nm. The polydispersity index (PDI) of the samples, which refers to the broadness of the particle size distribution, was acceptable as well (≤0.2), and the zeta potential, which reflects the surface charge of particles in suspension, fluctuated roughly between −5 mV and +5 mV in water at 25 °C.

**Table 2:** Characteristics of the non- and pictilisib-loaded LNPs (n = 3 replicates) (cargo load of 1 % w/w of the total lipid and stabilizer) (d_h_ = hydrodynamic diameter, PDI = polydispersity index, ζ = zeta potential, LC = loading capacity, EE = encapsulation efficiency).

Composition	Nomenclature	Cargo	d_h_ (nm) ± SD	PDI ± SD	ζ (mV) ± SD	LC (%) ± SD	EE (%) ± SD
**DMG-PEG2k, lecithin**	PEG-Lipid 1	–	234 ± 14	0.2 ± 0.03	−1 ± 6	–	–
		Pictilisib [1 wt%]	232 ± 25	0.1 ± 0.05	−5 ± 4	0.43 ± 0.17	43.1 ± 17.1
**ALC-0159, lecithin**	PEG-Lipid 2	–	228 ± 7.0	0.17 ± 0.03	−3 ± 1	–	–
		Pictilisib [1 wt%]	225 ± 8.0	0.14 ± 0.04	−2 ± 1	0.55 ± 0.06	55.2 ± 5.5
**PEtOx_20_-Lipid, lecithin**	POx-Lipid	–	205 ± 2.0	0.15 ± 0.02	1 ± 3	–	–
		Pictilisib [1 wt%]	204 ± 7.0	0.19 ± 0.01	1 ± 1	0.69 ± 0.17	69.5 ± 17.0

**Fig. 3:**
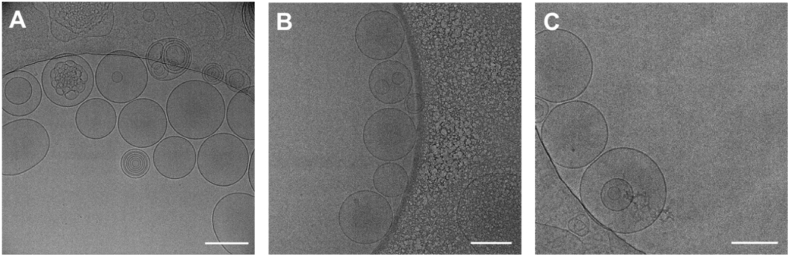
Cryo-TEM images of pictilisib-loaded LNPs based on (A) DMG-PEG2k (PEG-Lipid 1), (B) ALC-0159 (PEG-Lipid 2), and (C) PEtOx_20_-Lipid (POx-Lipid). Scale bars represent 200 nm.

Cryogenic transmission electron microscopy (cryo-TEM) analysis revealed that the LNPs exhibited a spherical vesicular structure with a size distribution consistent with the findings reported by DLS (Fig. 3). The particles showed a diffuse core, indicative of successful encapsulation and distribution of the drug inside. The presence of a lipid shell was visible, suggesting structural integrity. These findings were consistent among all three lipids tested. In addition to predominantly unilamellar vesicles, some particles displayed more complex morphologies, including multilamellar structures with multiple concentric lipid bilayers, core-in-core vesicles with smaller vesicles enclosed within larger ones, and multicompartment vesicles featuring possible internal aqueous compartments. These structural features are characteristics of LNPs formed through self-assembly and are frequently observed in hybrid or vesicular systems [67,82].

As for the drug encapsulation, the PEGylated LNPs achieved a loading capacity of approximately 0.43–0.55 % (wt%), indicating the weight of the encapsulated pictilisib relative to the total LNP weight. This loading increased to approximately 0.7 % in the POx-Lipid. Considering the amphiphilic nature of pictilisib and difficult encapsulation potential, the deviation in loading among different samples was observed. However, the loading achieved was sufficient for biological characterization. It is important to note that no drug precipitates have been observed in these formulations in the electron microscopy images in comparison to the polymeric nanoparticles (Fig. 3).

To facilitate uptake studies, LNPs were formulated with the fluorescent dye 1,1′-dioctadecyl-3,3,3′,3′-tetramethylindocarbocyanine (DiI), enabling their visualization and tracking. These particles successfully encapsulated the dye by following the same method as for pictilisib encapsulation, and the characteristics of the LNPs are analogous to those of the drug LNP ones, as shown in Table S2. The only notable exception was the zeta potential of the LNPs, which ranged between +20 and +25 mV, most likely due to the interaction between the dye and the components of the particle formulation.

In contrast to currently available strategies, this work presents a novel POx-Lipid LNP that incorporates POx as a biocompatible alternative to PEG, thereby addressing ongoing concerns regarding PEG-associated immunogenicity and stability. The resulting POx-Lipid-based formulation constitutes a new class of materials, distinct from the lipid systems employed in currently approved COVID-19 vaccines and provides a versatile alternative for future LNP designs. Importantly, the developed platform enables the successful encapsulation of pictilisib, a clinically relevant yet notoriously difficult-to-formulate PI3K inhibitor, thereby reviving its potential as an antiviral therapeutic. Overall, this formulation strategy represents a unique and innovative approach, integrating a challenging small-molecule PI3K inhibitor into an optimized nanocarrier platform tailored for antiviral applications.

### 3.2 Free pictilisib, non- and pictilisib-loaded LNPs have different impact on the cytotoxicity

**Fig. 4:**
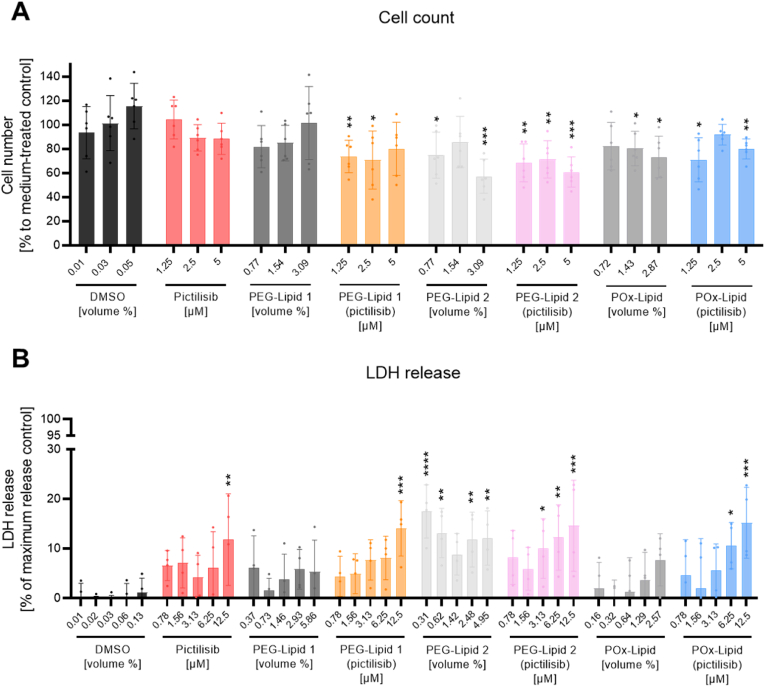
Pictilisib, non-and pictilisib-loaded LNPs have varying impact on the cytotoxicity. After 24 h of cultivation, Calu-3 cells were treated with the indicated concentrations of free pictilisib, PEG-Lipid 1 and 2 (pictilisib), POx-Lipid (pictilisib) and the corresponding volumes in % of the solvent control (DMSO), PEG-Lipid 1 and 2 and POx-Lipid for 24 h. (A) Cells were trypsinated and counted. Medium-treated samples were set to 100 %. The data represent the mean ± SD of three independent experiments with two technical replicates. Statistical significance was determined by one sample *t*-test with the hypothetical mean set to 100. ∗*p* < 0.05, ∗∗*p* < 0.01, ∗∗∗*p* < 0.001. Comparisons without asterisks did not reach statistical significance. (B) Supernatants were used to measure LDH release. Lysis buffer samples represent maximum LDH release control and were set to 100 %, while samples treated with medium alone served as spontaneous LDH release control and were set to 0 %. The data represent the mean ± SD of four independent experiments with three technical replicates. Statistical significance was determined by two-way ANOVA with Dunnett's multiple comparisons test. ∗*p* < 0.05, ∗∗*p* < 0.01, ∗∗∗*p* < 0.001. Comparisons without asterisks did not reach statistical significance.

For further use of the produced LNPs in cell culture analyses, they were first investigated for their cytotoxic properties. Initially, Calu-3 cells were utilized, which serve as a cell-culture model system for studying pulmonary infections caused by viruses. These cells are derived from a human lung adenocarcinoma and exhibit several characteristics of the bronchial epithelium, including mucin production, cilia expression, and the formation of tight junctions [83,84]. Therefore, Calu-3 cells are frequently used in toxicant research [85], drug delivery [86], and viral infections [87]. To establish a concentration range in which pictilisib and the LNPs do not exhibit cytotoxic effects, cell count as well as LDH assays were performed (Fig. 4). To ensure accurate comparisons, the amount of the pictilisib solvent (DMSO) and the non-loaded LNPs were adjusted to correspond to the same volumes of suspension administered to the substance-treated cells. For DMSO, the amount is expressed as a percentage, representing the volume of DMSO relative to the medium. Similarly, the non-loaded LNP amount indicates the proportion of pictilisib-loaded LNP suspension in the medium in volume %. To ensure comparability, LNP volumes were adjusted based on pictilisib content, standardizing treatment conditions across experimental groups. Notably, the different volumes of the various LNPs added to the cells were the result of the different loading efficiencies of encapsulated pictilisib.

For the cell count assays, cells were treated for 24 h with the indicated concentrations or volumes of substances and controls. Following the treatment and incubation, the cells were counted, and their cell numbers were normalized against a medium-treated control (Fig. 4A). Treatment with DMSO revealed no effect on the cell number in a volume of up to 0.05 %, which corresponds to 5 μM pictilisib. Likewise, treatment with pictilisib had no significant impact on the cell number of Calu-3 cells at concentrations of 1.25–5 μM. However, the addition of the three LNPs (PEG-Lipid 1, PEG-Lipid 2, and POx-Lipid) showed different effects on the cell number. PEG-Lipid 1 did not affect cell viability, while PEG-Lipid 2 addition appeared to have a significant cytotoxic effect, even though both were treated with the same volume of LNPs. POx-Lipid treatment demonstrated a slight impact on cell viability, reaching 80 % at volumes of 1.4 % and 2.9 %, which corresponds to 2.5 μM and 5 μM pictilisib. Nevertheless, this level remains within the acceptable range as defined by DIN EN ISO 10993-5. Regarding the pictilisib-loaded LNPs, treatment with PEG-Lipid 1 (pictilisib) and POx-Lipid (pictilisib) yielded comparable cell counts across the concentration range, from 71 % (PEG-Lipid 1 (pictilisib)) and 73 % (POx-Lipid (pictilisib)) at 1.25 μM, to 80 % at 5 μM, respectively. In contrast, the cytotoxic effect observed after adding PEG-Lipid 2 was more pronounced in PEG-Lipid 2 (pictilisib)-treated cells, with an overall cell survival rate decreasing from 68 % (1.25 μM) to 60 % (5 μM). To further validate the biological effects of free pictilisib, pictilisib-loaded and non-loaded LNPs on Calu-3 cells, LDH assays were performed after 24 h of treatment (Fig. 4B), as LDH is a marker released by cells undergoing membrane damage and cell death. DMSO addition exhibited no impact on LDH release, while treatment with pictilisib induced a slight increase in LDH release, which became significant at a concentration of 12.5 μM, with a 12 % LDH release compared to the control with maximum LDH release. Non-loaded PEG-Lipid 1 and POx-Lipid caused only minor alterations in LDH release over the volume range. However, PEG-Lipid 2 treatment resulted in a significant increase in LDH release across all added volumes. PEG-Lipid 2 (pictilisib) treatment provoked a similar effect on LDH release. This LDH release could be explained by the known phenomenon of lipid-containing formulations interacting with cellular membranes, promoting their destabilization [46,88]. In the presence of PEG-Lipid 1 (pictilisib) and POx-Lipid (pictilisib) similar patterns to pictilisib alone were observed, with concentrations up to 6.25 μM proving to be well-tolerated by the cells. In general, a slight elevation in LDH levels observed is believed to be a consequence of transient lipid-lipid interactions at the cell membrane interface, rather than genuine membrane disruption [89,90].

By taking the results from both cytotoxicity assays into consideration, PEG-Lipid 2 on its own as well as PEG-Lipid 2 (pictilisib) exhibited substantial impacts on cell viability across the tested concentrations and volume ranges. Therefore, they were not included in further experiments. It could be speculated that this is due to the fact that ALC-0159 (PEG-Lipid 2) has a stable amide bond so it is less prone to degradation while the ester moiety in DMG-PEG (PEG-Lipid 1) may be more easily hydrolyzed. The diminished cytotoxicity of the POx-Lipid can be explained by the fact that the POx itself has a linker that contains an ester bond. As such, treatment with PEG-Lipid 1 (pictilisib) and POx-Lipid (pictilisib) had only minor effects on cell viability at pictilisib concentrations up to 5 μM after 24 h of treatment. This concentration is also tolerable when treating cells with pictilisib alone. The low cytotoxic impact of pictilisib may be due to the diverse roles of the PI3K signaling pathway [14]. Notably, PEG-Lipid 1 and POx-Lipid showed minimal impact on cytotoxicity when administered at volumes up to 5 %, correlating to a pictilisib concentration of about 12 μM, underscoring their suitability as effective vehicles for drug delivery.

### 3.3 Pictilisib-loaded LNPs exert antiviral effects through PI3K inhibition

To investigate the cellular uptake of PEG-Lipid 1 and POx-Lipid, fluorescence plate reader analysis and confocal imaging were performed (Fig. S5). Therefore, DiI-loaded LNPs were prepared, to visualize the uptake of LNP into Calu-3 cells. The fluorescence intensity of PEG-Lipid 1 (DiI) and POx-Lipid (DiI) dilutions was measured beforehand. This revealed a slightly higher fluorescence signal for the POx-Lipid (DiI), indicating a higher DiI concentration. This trend was consistently observed across all dilutions (Fig. S5A). A concentration of 0.1 mg mL^−1^ of PEG-Lipid 1 (DiI) and POx-Lipid (DiI) was administered to Calu-3 cells, and internalization was examined within a timeframe from 1 to 24 h (Fig. S5B) or after 4 h (Fig. S5C) and 24 h (Fig. S5D). As depicted in Fig. S5B, the fluorescence intensity increased progressively from 1 to 24 h for both LNPs, with the observed increase being slightly more pronounced for POx-Lipid (DiI), probably due to the fact that the initial DiI concentration was already lower in the PEG-Lipid (DiI) (Fig. S5A). These results confirm the successful internalization of the PEG-Lipid 1 and POx-Lipid into Calu-3 cells, validating their uptake and demonstrating the effectiveness of the formulation for cellular delivery.

**Fig. 5:**
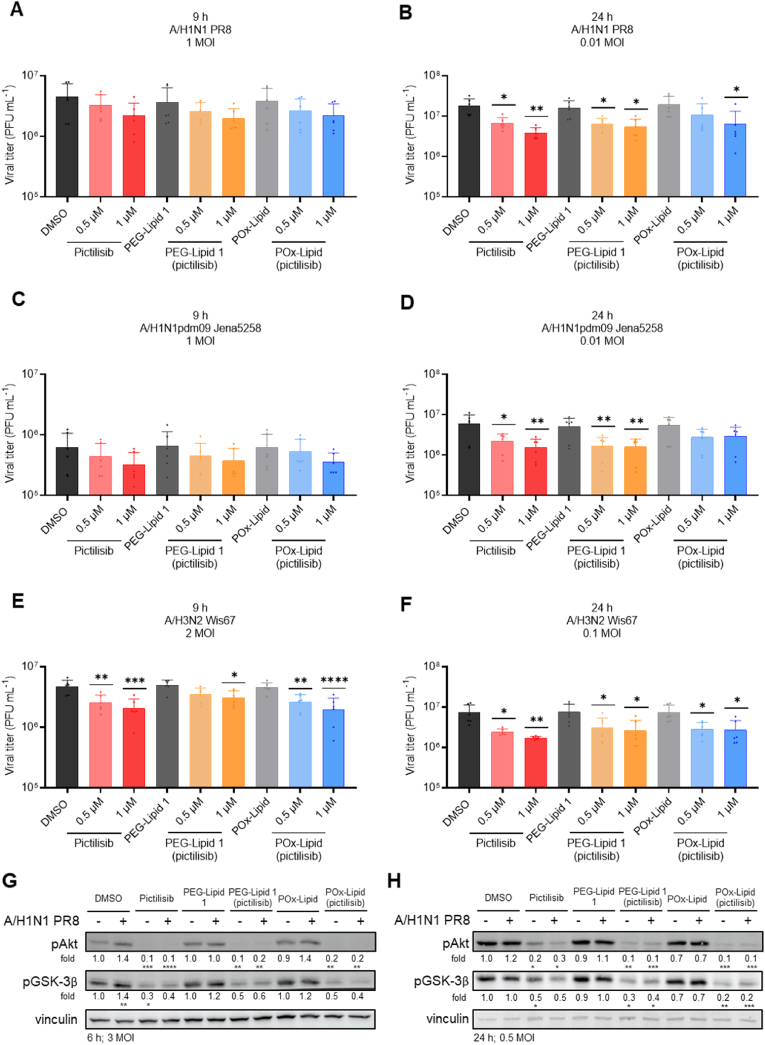
Treatment with pictilisib and pictilisib-loaded LNPs inhibits the replication of different IAV strains and IAV-induced PI3K signaling. Calu-3 cells were pretreated for 1 h with 0.5 μM and 1 μM (A–F) or only 1 μM (G, H) of the indicated substances (pictilisib, PEG-Lipid 1 (pictilisib), POx-Lipid (pictilisib)) or controls (DMSO, PEG-Lipid 1, POx-Lipid) and were subsequently infected with the indicated IAV strains (A/H1N1 PR8 (A, B, G, H), A/H1N1pdm09 Jena5258 (C, D) or A/H3N2 Wis67 (E, F)) and multiplicity of infection (MOI) for 30 min. After that, virus solution was removed and cells were treated again with 0.5 μM or 1 μM of the substances or corresponding volumes of the controls. At 9 h (A, C, E) or 24 h p.i. (B, C, F) progeny virus titers (plaque forming units (PFU mL^−1^)) were determined by plaque assay. (A–F) The mean +SD of three independent experiments with two technical replicates is depicted. Statistical significance was determined using one-way ANOVA with Dunnett's multiple comparisons test. ∗*p* < 0.05, ∗∗*p* < 0.01, ∗∗∗*p* < 0.001, ∗∗∗∗*p* < 0.0001. Comparisons without asterisks did not reach statistical significance. (G, H) Cell lysates were collected after 6 h (G) or 24 h (H) p.i. for Western blot analysis. PI3K-mediated signaling was indirect detected via phosphorylation of Akt and GSK-3β. (G, H) Data represent one representative out of three independent experiments. The densitometry is represented as the mean fold protein expression of three independent experiments relative to DMSO-treated uninfected samples, normalized to vinculin and set to 1 (Fiji ImageJ 1.54i quantification). Statistical significance was determined by one-sample *t*-test comparing to a hypothetical mean of 1. ∗*p* < 0.05, ∗∗*p* < 0.01, ∗∗∗*p* < 0.001. Comparisons without asterisks did not reach statistical significance.

To assess the antiviral effect of pictilisib-loaded LNPs, single-cycle and multi-cycle viral replication experiments were conducted, with free pictilisib included as a control due to its previously established antiviral activity [34]. The concentrations of 0.5 μM and 1 μM of pictilisib used in these experiments were selected based on their negligible effects on cell viability, which were observed in previous cytotoxicity assays. The amount of DMSO and non-loaded LNPs were adjusted to match the administered volume of 1 μM pictilisib or pictilisib-loaded LNPs. Calu-3 cells were pretreated with the respective substances or LNPs for 1 h, after that the supernatants were removed and cells were infected with one of the three IAV strains (A/H1N1 PR8, A/H1N1pdm09 Jena5258, and A/H3N2 Wis67) at the indicated multiplicity of infection (MOI). The initial infectious doses were chosen based on previous infection experiments, which demonstrated different replication efficiencies of the IAV strains in Calu-3 cells (Fig. 5). After an incubation period of 30 min, cells were washed and retreated. The supernatants were collected to determine viral load using plaque assays 9 h (Fig. 5A–C, E) and 24 h (Fig. 5B–D, F) p.i. After 9 h, viral titers in cells treated with pictilisib or pictilisib-loaded LNPs, revealed slight reductions when infected with the strains A/H1N1 PR8 and A/H1N1pdm09 Jena5258, while a significant reduction was detected in A/H3N2 Wis67-infected cells. In contrast, a significant reduction in viral titers was visible 24 h p.i., regardless of the viral strain. This reduction was observed at both 0.5 μM and 1 μM, with a stronger decrease after treatment with 1 μM. Importantly, non-loaded LNPs exhibited no impact on viral titers 9 h and 24 h p.i. and were consistent with the DMSO control. The results suggest that the antiviral properties of pictilisib-loaded LNPs extend beyond their efficacy against A/H1N1 subtypes, as they also effectively reduced the viral load during infection with the A/H3N2. In addition, other studies have demonstrated the antiviral potential of pictilisib against both human and avian IAV isolates [34,35]. This supports the conclusion that pictilisib exhibits pan-antiviral activity, as it is not only effective against IAV strains circulating in humans but also against strains found in poultry, which is comparable to other RNA viruses such as SARS-CoV-2 [91,92]. Furthermore, earlier studies with PI3K inhibitors demonstrated antiviral activity against not only various IAV strains but also against IBVs in different cell lines [23,27]. In the present study, Calu-3 cells were used as an *in vitro* model, as the primary objective was to compare the antiviral properties of encapsulated pictilisib to those of free pictilisib during IAV replication.

To investigate the effect of pictilisib-loaded LNPs on IAV-induced activation of PI3K-mediated signaling, protein phosphorylation and activation within the first and multicyclic replication were examined. Thus, cell lysates were collected for Western blot analysis in infection setups lasting 6 h (Fig. 5G) or 24 h (Fig. 5H). Viral infection led to an upregulation of Akt phosphorylation at serine 473 (Ser473) as well as increased levels of pGSK-3β at serine 9 (Ser9) at both time points. This phenomenon is well-documented and strongly depends on the activation of the PI3K signaling pathway [24,27]. Treatment with pictilisib and pictilisib-loaded LNPs resulted in a significant reduction in the IAV-induced pAkt and pGSK-3β signals. Notably, the inhibition of IAV-induced Akt and GSK-3β phosphorylation appears to be more pronounced after 6 h of treatment with free pictilisib (Fig. S6). However, after 24 h of infection, the protein expression levels of pAkt and pGSK-3β were significantly reduced following the administration of PEG-Lipid 1 (pictilisib) and POx-Lipid (pictilisib). This observation suggests that LNP-encapsulated pictilisib is not immediately released and available in contrast to the immediate presence of the free compound, but rather exhibits a depot effect, resulting in sustained drug release over a longer period of time [93]. This delayed but sustained release is further supported by the internalization studies, which showed that the intracellular accumulation of LNPs continued to increase even after 24 h compared to 4 h (Fig. S5B and C).

The results demonstrate that treating IAV-infected Calu-3 cells with pictilisib-loaded LNPs at non-toxic concentrations effectively reduces viral load and replication by modulating the PI3K signaling pathway *in vitro*. Both pictilisib-loaded PEG-Lipid 1 and pictilisib-loaded POx-Lipid exhibit similar antiviral efficacy to free pictilisib.

### 3.4 In the presence of pictilisib-loaded LNPs IAV-induced cytokine and chemokine production is reduced *in vitro*

**Fig. 6:**
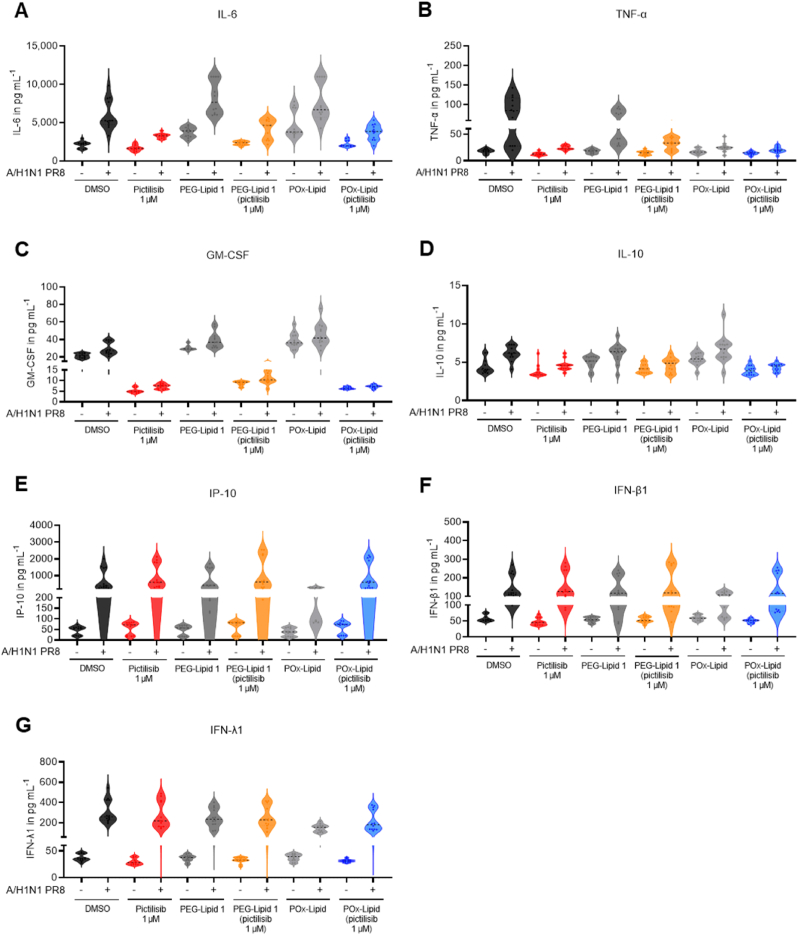
Treatment with pictilisib and pictilisib-loaded LNPs affects the IAV-induced cytokine and chemokine production at 24 h p.i. *in vitro*. Calu-3 cells were pretreated with 1 μM pictilisib, PEG-Lipid 1 (pictilisib) or POx-Lipid (pictilisib) or corresponding volumes of the controls (DMSO, PEG-Lipid 1, POx-Lipid) for 1 h prior to infection and were after that infected with 0.5 MOI of A/H1N1 PR8 for 30 min. Virus solution was removed after that and substances and respective controls were added again, and cells were incubated for further 23.5 h. After that supernatants were collected, and the amount of the indicated cytokines and chemokines was detected using flow cytometry (LEGENDplex). Diagrams show three independent experiments with technical duplicates.

Besides the production of new progeny virus particles, IAVs also trigger the release of pro- and anti-inflammatory cytokines upon infection of lung epithelial cells [94]. Although these cytokines are crucial for the initiation of immune defense mechanisms, they also can contribute to cell damage [95]. To assess the disease progression, the expression of various cytokines and chemokines was analyzed by flow cytometry 24 h p.i. Interleukin (IL)-6, tumor necrosis factor (TNF)-α, granulocyte-macrophage colony-stimulating factor (GM-CSF), IL-10, interferon γ-induced protein (IP)-10, interferon (IFN)-β1, and IFN-λ1 were significantly upregulated upon IAV infection (Fig. 6). Concomitantly, IL-6, TNF-α, IL-10 and GM-CSF were reduced by treatment with pictilisib and LNP (pictilisib) during IAV infection (Fig. 6A, B, C, D). The levels of IL-1β, IL-12p70, IFN-α2, IFN-λ2/3, IFN-γ1 were not affected, neither by IAV infection nor the treatment applied (Fig. S7). Since cytokine and chemokine measurements were performed on cells infected for 24 h, which represents multi-cyclic replication, accumulating effects due to reduced viral replication seems likely. While the results of cytokine and chemokine levels presented here, partially differ from the results obtained with pictilisib-mediated effects in IAV infection *in vivo* [34], due to the fact that cytokines secreted by immune cells differ from those of epithelial cells, our results are consistent with the findings obtained with the PI3K inhibitor LY294002, which suppressed IAV-induced IL-6 production in A549 cells [96]. Although the expression of IP-10 is strongly dependent on IAV replication, no significant differences were observed between the pictilisib treatment and the solvent control which is consistent with previous results [34]. Overall, pictilisib-loaded LNPs exhibited similar behavior to free pictilisib. Interestingly, in presence of the non-loaded POx-Lipid, increased levels of IL-6, TNF-α, IFN-β1, and IP-10 have already been observed (Fig. 6A, B, E, F). These results suggest that LNPs themselves have the potential to influence the immune response, a phenomenon that is well-documented for mRNA-LNP vaccines [97,98]. Accordingly, recent research has demonstrated that the immune response to LNPs is driven by the activation of toll-like receptors and other pattern recognition receptors that specifically identify the lipid components within the LNPs [98,99].

### 3.5 In presence of pictilisib-loaded LNPs, IAV infection is inhibited in an *ex vivo* mouse lung model

The initial testing of antiviral agents primarily relies on cell-based assays that focus on individual host factors involved in the response to infection. These studies are necessary to obtain initial evidence of the antiviral and anti-inflammatory potential of test substances. However, such approaches have limitations and carry considerable risks for further animal studies or clinical trials. Consequently, drug development relies on more complex model systems. However, animal testing is not only costly but also requires the use of large numbers of mice, which raises significant ethical concerns and must be minimized. The use of lung slices offers a promising alternative with up to 150 slices generated from a single mouse lung, depending on the chosen section thickness, thereby significantly reducing animal use [100]. Lung slices consist of multiple cell types and maintain physiological and functional relationships, which closely mimic lung morphology and functionality. To validate the efficacy of pictilisib-loaded LNPs in a more complex model, *ex vivo* lung slices from C57BL/6JRj mice were used. These mice are susceptible to IAV infection after intranasal administration, as confirmed by our own *in vivo* experiments [34,101] and others [102]. The *ex vivo* slices remain viable and infectible in medium for at least three days after preparation [100,103]. Based on a kinetic experiment measuring viral titers at 10, 24, 48, and 72 h p.i. (Fig. S9), a 48 h time point was selected for the antiviral efficacy experiments, as it represents the peak of viral replication. This replication peak at 48 h p.i. in *ex vivo* lung slices is consistent with previous observations for results obtained using A/H1N1pdm09 Jena5258 virus isolate in the lungs of infected mice [63]. This time point therefore provides an optimal window to assess antiviral effects while minimizing confounding factors such as late-stage viral decline or tissue damage. For *ex vivo* mouse lung infection experiments, POx-Lipid (pictilisib) was selected as it demonstrated comparable antiviral efficacy to PEG-Lipids *in vitro*. Furthermore, POx-Lipids were preferred because, unlike PEG-Lipids, no hypersensitivity reactions have been reported in association with their use until now [53,55]. To confirm the internalization of POx-Lipids into the lung tissue, confocal microscopy images were taken, which provided clear evidence of POx-Lipid (DiI) uptake after 24 h (Fig. S8).

**Fig. 7:**
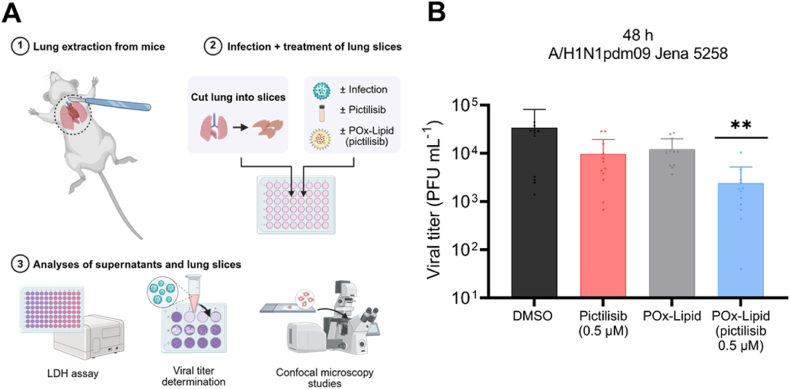

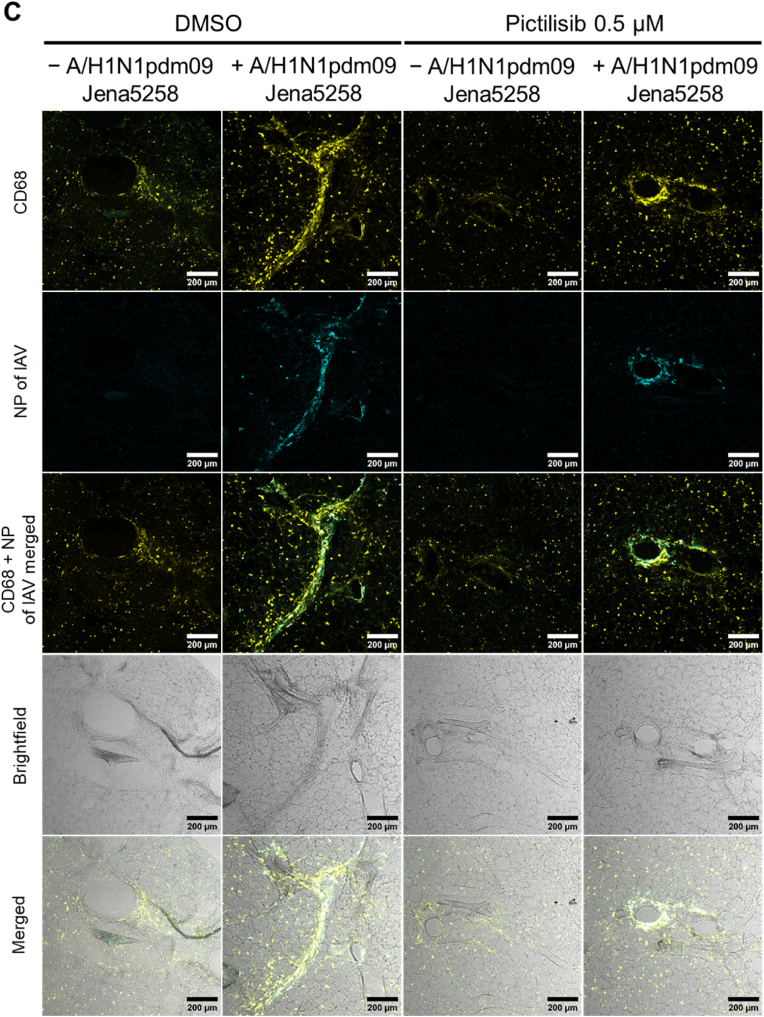

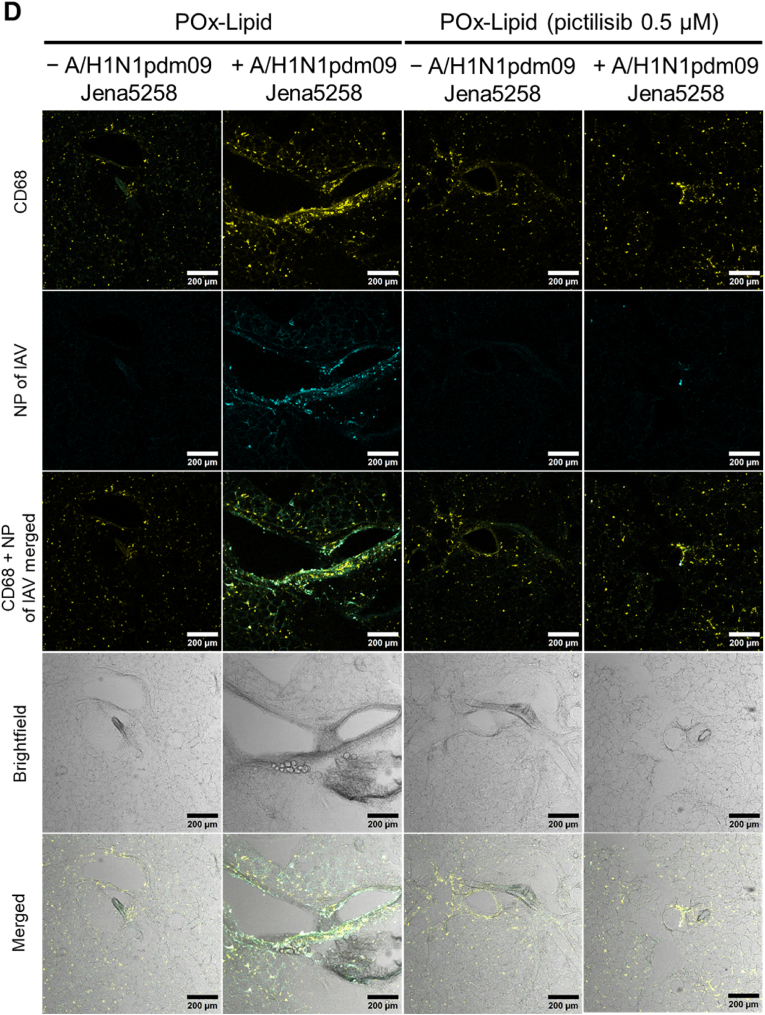
In the presence of pictilisib and POx-Lipid (pictilisib) IAV replication is reduced in *ex vivo* mouse lung slices. (A) Mouse lung was extracted, cut into slices, infected with A/H1N1pdm09 Jena5258 for 3 h (B–D) or left uninfected (C–D) and afterwards treated with 0.5 μM pictilisib, POx-Lipid (pictilisib) or respective volumes of the controls (DMSO, POx-Lipid) for 45 h. (B) At 48 h p.i. supernatants were used to determine progeny virus titers (PFU mL^−1^) by plaque assay. The mean +SD of three independent mice with four technical replicates is depicted. Statistical significance was determined using one-way ANOVA with Dunnett's multiple comparisons test. ∗∗*p* < 0.01. Comparisons without asterisks did not reach statistical significance. (C–D) Alveolar macrophages were stained with a rat anti-CD68 primary antibody and an anti-rat Cy3 secondary antibody (yellow). Viral nucleoprotein (NP) is stained with a mouse anti-IAV NP primary and an anti-mouse AF488 secondary antibody (cyan). Lung structure is shown through brightfield imaging. Scale bars represent 200 μm. Depicted is one out of three independent mice. (For interpretation of the references to colour in this figure legend, the reader is referred to the Web version of this article.)

The mouse lung slices were infected with the A/H1N1pdm09 Jena5258 strain [34]. Following the infection, pictilisib and POx-Lipid (pictilisib) as well as DMSO and non-loaded POx-Lipid were applied, and the samples were incubated for a total of 48 h (Fig. 7A). The pretreatment step, as used in the *in vitro* experiments, was omitted to better reflect the clinical situation, as treatment would only begin after an infection had occurred. The results show that in presence of pictilisib viral titers are reduced, an effect that is further enhanced by encapsulation in the POx-Lipid (Fig. 7B). Even the addition of POx-Lipid alone resulted in a slight reduction in viral titers, potentially due to its interaction with cellular membranes, which may alter the local microenvironment and inhibit viral replication. Importantly, this reduction is not attributable to increased cytotoxicity, as LDH levels were comparable across all compound-untreated samples (Fig. S10). Notably, IAV infection induced LDH release in all samples, with slightly lower levels observed in the pictilisib- and POx-Lipid (pictilisib)-treated conditions (Fig. S10).

Confocal imaging shows NP (Fig. 7C and D; Fig. S11) and M2 protein (Fig. S12) of IAV, serving as indicators of viral infection. CD68 staining indicated the presence of alveolar macrophages, while brightfield imaging illustrates the structure of the mouse lung tissue. The findings indicate that treatment with pictilisib effectively inhibits IAV infection, with an even greater reduction observed when using POx-Lipid (pictilisib). This observation is supported by quantitative analysis of NP and M2 fluorescence signal intensities, presented in Figure S11 and Figure S12C, respectively. Alveolar macrophages are actively recruited to the site of infection, which is predominantly the bronchial epithelium. Interestingly, across all infection samples, both epithelial cells lining the bronchi and alveolar macrophages show significant infection, whereas the submucosal cell layers remain largely unaffected. This phenomenon has been observed in previous studies using *ex vivo* lung models, where variations in sialic acid (SA) linkage densities influence virus binding [104]. The bronchial epithelium tends to have a higher concentration of α-2,6-linked SAs, which facilitates attachment for human or other mammalian IAV strains. This density decreases further down the respiratory tract, affecting viral interactions accordingly [105,106]. Similar patterns have been reported in different species, highlighting variations in the prevalence of α-2,6-linked and α-2,3-linked SAs across lung tissue [107].

Overall, the results demonstrate the superior effect of encapsulated pictilisib in an *ex vivo* lung model. The advantage of LNP encapsulation was also reported in another study, where nebulized PEG-LNPs containing therapeutic mRNA were successfully administered *in vivo* to the lungs, showing enhanced performance [108]. Inhalation presents a particular promising route of administration, as it targets the lungs directly with lower risk of systemic distribution. The safety and optimal duration of inhalation-based treatment require thorough evaluation to fully assess the balance between its therapeutic advantages and potential risks. These findings, both from our study and the literature, underscore promising improvements in drug delivery and stability in more complex systems. However, further studies are necessary to determine whether the encapsulation of PI3K inhibitors in LNPs achieves comparable enhancements in more complex systems, such as *in vivo* models.

## 4 Conclusion

The treatment of IAV infection traditionally relies on drugs that directly target the virus. This often leads to the significant challenge of developing high levels of resistance. To address this problem, repurposing of existing drugs that target specific intracellular signaling pathways essential for viral replication has become an important area of research. However, many of these drugs exhibit high toxicity or low bioavailability, limiting their successful application in humans. In this study, PEG- and POx-Lipids were utilized in LNP formulations as a novel antiviral drug delivery platform. PEG-LNPs are already widely used but are associated with allergenic concerns due to their presence in various daily products. Hence, POx-Lipids were introduced as an alternative for comparative analysis. To assess its efficacy during infection, pictilisib, a PI3K inhibitor, known for its activity against IAV infections, was encapsulated in these LNPs. *In vitro* experiments demonstrated that pictilisib-loaded LNPs were as effective against IAV infections as free pictilisib, exhibiting both antiviral and anti-inflammatory properties. *Ex vivo* experiments revealed that the novel POx-Lipid nanocarriers show a more potent effect on the infection than free pictilisib. These findings indicate that this platform offers improved pharmacokinetics, with the potential to enhance drug stability and enable targeted delivery to infected tissues. The PEG-Lipid- and POx-Lipid-based LNPs described in this study, especially considering the additional enhanced antiviral performance of the POx-Lipid LNPs in *ex vivo* mouse lung slices, offer a strong translational promise due to its biocompatible material composition that is adaptable to different small-molecule cargos, including clinically relevant inhibitors such as pictilisib. Moreover, the use of POx as a PEG alternative could mitigate concerns related to PEG immunogenicity, improving long term safety. Nonetheless, we acknowledge several limitations that warrant further investigation in a future study, such as the need for *in vivo* evaluation of the pharmacokinetics, biodistribution, as well as antiviral efficacy. Comprehensive safety and immunogenicity assessments should be well conducted prior to clinical translation. Nonetheless, this represents a promising and valuable area of research that warrants further exploration.

## CRediT authorship contribution statement

**Josefine Schroeder:** Writing – review & editing, Writing – original draft, Visualization, Software, Methodology, Investigation, Formal analysis, Data curation, Conceptualization. **Jana Ismail:** Writing – review & editing, Writing – original draft, Visualization, Software, Methodology, Investigation, Formal analysis, Data curation, Conceptualization. **Caroline T. Holick:** Writing – review & editing, Writing – original draft, Visualization, Software, Methodology, Investigation. **Johannes Jungwirth:** Writing – review & editing, Validation, Investigation. **Laura Klement:** Writing – review & editing, Validation, Investigation. **Stephanie Hoeppener:** Writing – review & editing, Software, Investigation. **Christian Kosan:** Writing – review & editing, Resources. **Michaela Schmidtke:** Writing – review & editing, Resources. **Bettina Löffler:** Writing – review & editing, Supervision, Resources. **Christine Weber:** Writing – review & editing, Validation, Supervision, Resources. **Ulrich S. Schubert:** Writing – review & editing, Supervision, Project administration, Funding acquisition. **Carsten Hoffmann:** Writing – review & editing, Supervision, Resources. **Stephanie Schubert:** Writing – review & editing, Writing – original draft, Supervision, Project administration, Conceptualization. **Christina Ehrhardt:** Writing – review & editing, Writing – original draft, Supervision, Project administration, Conceptualization.

## Funding

This research was funded by the 10.13039/501100001659German Research Foundation (CRC 1278, project number 316213987, projects D02, Z01) and further supported by the 10.13039/100021130Federal Ministry for Economic Affairs and Climate Action (BASE-Lipid, 16LP401003). The cryo-TEM investigations were performed at the Electron Microscopy facilities of the Jena Center for Soft Matter (JCSM). The Titan Krios G4 was funded by the Thüringer Ministerium für Bildung, Wissenschaft und Kultur (TMBWK). The SEM facilities of the Jena JCSM were established with a grant from the German Research Foundation (10.13039/501100001659DFG). The MALDI-TOF mass spectrometer (rapifleX) was funded by Tühringer Aufbaubank (TAB; funding ID: 2016 IZN 0009). Open access funding enabled and organized by Projekt DEAL.

## Declaration of competing interest

The authors declare that they have no known competing financial interests or personal relationships that could have appeared to influence the work reported in this paper.

## Data Availability

Data will be made available on request.
