# Supplementary material for: Advancing influenza virus treatment: in vitro and ex vivo studies of PI3K inhibitor-loaded lipid nanoparticles
Source: Mater Today Bio. 2025 Nov 26;35:102587. doi: 10.1016/j.mtbio.2025.102587 (PMC12704091; doi:10.1016/j.mtbio.2025.102587)
Supplement: Multimedia component 1 [file mmc1.docx]

**Advancing influenza virus treatment: *in vitro* and *ex vivo* studies of PI3K inhibitor-loaded lipid nanoparticles**

Josefine Schroeder^a, 1^, Jana Ismail^b, c, 1^, Caroline T. Holick^b, c^, Johannes Jungwirth^a^, Laura Klement^d^, Stephanie Hoeppener^b, c^, Christian Kosan^e^, Michaela Schmidtke^a^, Bettina Löffler^f, c^, Christine Weber^b, c^, Ulrich S. Schubert^b, c, g, h^, Carsten Hoffmann^c, d^, Stephanie Schubert^b, c, 2,^ * and Christina Ehrhardt^a, c, 2,^ **

^a^ Section of Experimental Virology, Institute of Medical Microbiology, Center for Molecular Biomedicine (CMB), Jena University Hospital, Jena, Germany; josephine.schroeder@med.uni-jena.de; johannes.jungwirth@med.uni-jena.de; michaela.schmidtke@med.uni-jena.de
^b^ Laboratory of Organic and Macromolecular Chemistry (IOMC), Friedrich Schiller University Jena, Jena, Germany; jana.ismail@uni-jena.de; caroline.holick@uni-jena.de; s.hoeppener@uni-jena.de; christine.weber@uni-jena.de; ulrich.schubert@uni-jena.de
^c^ Jena Center for Soft Matter (JCSM), Friedrich Schiller University Jena, Jena, Germany
^d^ Institute of Molecular Cell Biology, Center for Molecular Biomedicine (CMB), Jena University Hospital, Friedrich Schiller University Jena, Jena, Germany; laura.klement@med.uni-jena.de; carsten.hoffmann@med.uni-jena.de

^e^ Department of Biochemistry, Center for Molecular Biomedicine (CMB), Friedrich Schiller University Jena, Jena, Germany; christian.kosan@uni-jena.de
^f^ Institute of Medical Microbiology, Jena University Hospital, Jena, Germany; bettina.loeffler@med.uni-jena.de

^g^ Helmholtz Institute for Polymers in Energy Applications Jena (HIPOLE Jena), Jena, Germany

^h^ Helmholtz-Zentrum Berlin für Materialien und Energie GmbH (HZB), Berlin, Germany

^1^ first authors.
^2^ equal contribution.
* Corresponding author: stephanie.schubert@uni-jena.de
** Corresponding author: christina.ehrhardt@med.uni-jena.de

**Supplementary Information:**

1. **Materials:**

2-Ethyl-2-oxazoline (EtOx; ≥99 %, Sigma-Aldrich, Darmstadt, Germany) was pre-dried over barium oxide (BaO; 90 %, Acros, Geel, Belgium) and distilled under inert conditions. Methyl tosylate (MeOTs; 97 %, Sigma-Aldrich, Darmstadt, Germany) was dried over calcium hydride (CaH_2_; Sigma-Aldrich, Darmstadt, Germany) and distilled under reduced pressure. Acetic acid (AcOH; ACS, Reag. Ph. Eur., VWR, Fontenay-sous-Bois, France), triethylamine (NEt_3_; Sigma-Aldrich, Darmstadt, Germany), 0.5 M sodium methoxide (NaOMe) in methanol (MeOH; ≥99 %, Sigma-Aldrich, Darmstadt, Germany), *N*-hydroxysuccinimide (NHS; Sigma-Aldrich, Darmstadt, Germany), 1-ethyl-3-(3-dimethylaminopropyl)carbodiimide (EDC; ≥97 %, Sigma-Aldrich, Darmstadt, Germany), ditetradecylamine (Evonik, Hanau, Germany), dimethylaminopyridine (DMAP; 99 %, abcr, Karlsruhe, Germany), succinic anhydride (Sigma-Aldrich, Darmstadt, Germany) and chloroform (CHCl_3_; anhydrous, >99 %, Sigma-Aldrich, Darmstadt, Germany) were used without further purification. Acetonitrile (CH_3_CN), methanol (MeOH) and dichloromethane (CH_2_Cl_2_) were dried in a solvent purification system (SPS 800; MBRAUN, Garching near Munich, Germany). Technical grade diethyl ether (Et_2_O) was used without further purification. Acetone (>99 %, extra pure, Acros, Geel, Belgium), acetonitrile (≥99.5 %; Roth, Karlsruhe, Germany) and ethyl acetate (ROTISOLV ≥99.9 %; Roth, Karlsruhe, Germany) were used in formulation preparation. Dialysis was performed using a Spectra/Por Biotech Cellulose Ester (CE) dialysis membrane with a molecular weight cut-off (MWCO) of 100 to 500 Da (Roth, Karlsruhe, Germany). Poly(d,l-lactic-*co*-glycolic acid) (PLGA) (acid terminated, Resomer RG 502 H, copolymer composition 50:50, molar mass of 7000 to 17,000 g mol^−1^) was purchased form Evonik (Darmstadt, Germany). Same PLGA was covalently coupled to DY635 to prepare PLGA-DY635 according to a previously published protocol (Press et al., 2014). The acetalation of dextran was achieved as well based on an earlier published procedure (Kauffman et al., 2012) (molar mass of original dextran = 10 kDa, Mw of AcDex 11.2 kDa, total degree of substitution (DS) 2.66, DS_cyclic_ = 1.95 and DS_acyclic_ = 0.71). The synthesis of the block copolymer of poly(2-ethyl-2-oxazoline) and poly(d-lactide) (PEtOx-*b*-PLA) has been reported previously (degree of polymerization (DP), DP(EtOx) = 18, DP(lLA) = 108; M_n_(NMR) = 15.5 kg mol^‑1^, Ð(SEC) = 1.30) (Stafast, 2024). Poly(ethylene glycol) (PEG) methyl ether-*block-*PLGA (PEG average M_n_ 5,000, PLGA M_n_ 5,000, lactide:glycolide 50:50; Merck, Darmstadt, Germany), (DSPE)-PEG(2000)-Carboxylic acid (Merck, Darmstadt, Germany), and soybean lecithin (EMD-Millipore, Darmstadt, Germany) were procured as well. Pictilisib (purity: 99.62 %) and 1,1′-dioctadecyl-3,3,3′,3′-tetramethylindocarbocyanine (DiI), a carbocyanine dye, were purchased from MedChemExpress (Sollentuna, Sweden).

1. **Instrumentation:**

*Nuclear magnetic resonance (NMR) spectroscopy*

Proton NMR (^1^H NMR) spectra were measured using a Bruker AC 300 MHz spectrometer. The measurement was performed at room temperature (RT) using CDCl_3_ as a solvent. The residual nondeuterated solvent signal was used as a reference. The spectra were baseline corrected using the software SpinWorks 4.

*Matrix assisted laser desorption / ionization time-of-flight mass spectrometry (MALDI TOF MS)*

MALDI TOF MS was measured on a rapifleX MALDI-TOF/TOF instrument from Bruker Daltonics equipped with a smartbeam™ 3D laser (355 nm wavelength). The spectra were measured in the positive reflector mode. *Trans*-2-[3-(4-*tert*-butylphenyl)-2-methyl-2-propenylidene]malononitrile (DCTB) was used as matrix, and sodium trifluoroacetate (NaTFA) was added as a doping salt. The recording was performed using manufacturer’s software flexControl 4.0. Evaluation and processing of the recorded spectra was done using manufacture’s software flexAnalysis 4.0 including baseline subtraction and external calibration using a 2,500 g mol^-1^ poly(methyl methacrylate) (PMMA) standard from Polymer Standard Services (PSS, Mainz, Germany).

*Size exclusion chromatography (SEC)*

SEC was measured on a Shimadzu system equipped with a CBM-20A system controller, a LC-10AD VP pump, a RID-10A refractive index detector, a SPD10AD VP UV detector, and a SDV linear S column from PSS at 40 °C using CHCl_3_:NEt_3_: *i*-PrOH (94:4:2) as eluent at a flow rate of 1 mL min^−1^. The calibration was made of polystyrene polymer standards of narrow molar mass distribution (Supplier: Polymer Standards Service (PSS), Mainz, Germany, M_p_ = 370 to 128,000 g mol^-1^).

*Scanning electron microscopy (SEM)*

Pictilisib precipitate was imaged by drop-casting 10 µL of sample on a mica substrate, leaving it to air dry, then coating it with a 4 nm platinum layer using a CCU-010 HV sputter (Safematic, Zizers, Switzerland). The measurements were conducted using a Sigma VP Field Emission Scanning Electron Microscope (Carl-Zeiss AG, Germany), which is equipped with an Inlens detector and operated at an accelerating voltage of 8 kV.


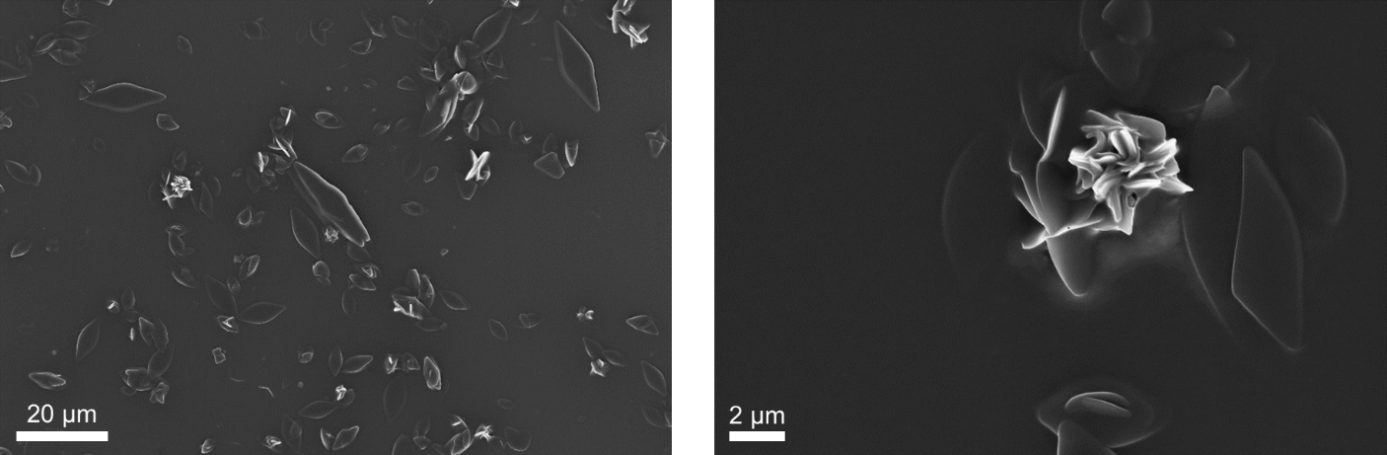


***Figure S1*.** SEM images of pictilisib as a drug precipitate. Scale bar represents 20 µm (left) or 2 µm (right).

1. **Synthesis procedure:**

PEtOx_20_-Lipid was synthesized in four steps, beginning with the cationic ring-opening polymerization of 2‑ethyl‑2‑oxazoline followed by end group modification reactions (Scheme S1). The characterization of the precursors can be found in Figure S2 and for the POx-Lipid in Figure S3.


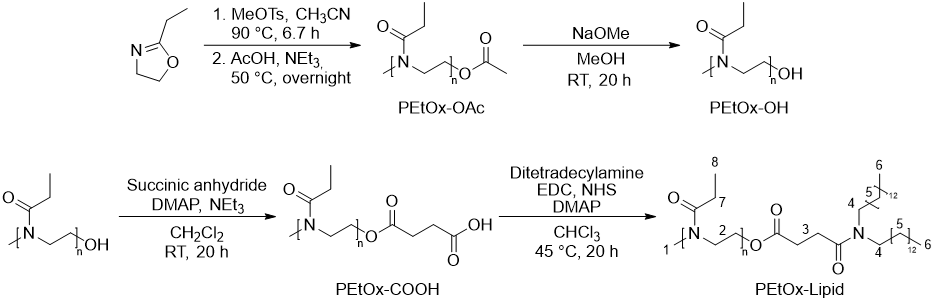


***Scheme S1*.** Schematic representation of the POx-Lipid synthesis.

## PEtOx_20_-OAc

In a pre-dried flask, which was degassed by a continuous argon stream, MeOTs (1.88 g, 10.1 mmol, 1 eq.) and 2-ethyl-2-oxazoline (20 g, 201.8 mmol, 20 eq.) were dissolved in anhydrous acetonitrile (30 mL). The reaction was stirred under reflux for 4.5 h and terminated by adding acetic acid (909 mg, 15 mmol, 1.5 eq.) and triethylamine (2.04 g, 20.2 mmol, 2 eq.). The reaction was allowed to stir at 50 °C overnight. The mixture was diluted with CHCl_3_ and washed twice with aqueous NaHCO_3_ and once with brine. The organic phase was dried over Na_2_SO_4_ and filtered. The solvent was removed under reduced pressure, and the residue was dried at 40 °C *in vacuo*. The product was obtained as a white powder (17.9 g, 87 %).

^1^H NMR (300 MHz, CDCl_3_): *δ* = 4.18 (br), 3.27 – 3.65 (br), 2.92 – 3.10 (m), 2.12 – 2.54 (m), 2.06 (br), 0.99 – 1.27 (br) ppm. DF = 86 %

SEC (CHCl_3_/*i*-PrOH/NEt_3_, RI detection, PS calibration): M_n_ = 3000 g mol^-1^, *Đ* = 1.06.

MALDI-TOF MS (DCTB + NaTFA): [CH_3_(C_5_H_9_NO)_2_C_2_H_3_O_2_+Na]^+^, *m/z* = 2276.56 observed.

## PEtOx_20_-OH

PEtOx-OAc (17.8 g, 9.08 mmol, 1 eq.) was dissolved in anhydrous MeOH (120 mL), and NaOMe (0.5 M in MeOH, 1.8 mL, corresponding to 0.91 mmol (0.1 eq.) NaOMe) was added under vigorous stirring, which was continued at RT overnight. MeOH was removed under reduced pressure and the residue was dissolved in CHCl_3_. The mixture was washed twice with NaHCO_3_ and brine. After drying of the organic phase over Na_2_SO_4_ and filtration, the solvent was removed under reduced pressure. The residue was then dissolved in dichloromethane and precipitated in cold Et_2_O (−80 °C). The solid was dried *in vacuo*. The product was obtained as a white powder (18.5 g, 105 %).

^1^H NMR (300 MHz, CDCl_3_): *δ* = 3.77 (br), 3.28 – 3.70 (br), 2.91 – 3.09 (m), 2.12 – 2.61 (m), 0.97 – 1.23 (br) ppm.

SEC (CHCl_3_/*i*-PrOH/NEt_3_, RI detection, PS calibration): M_n_ = 2740 g mol^-1^, *Đ* = 1.07.

MALDI-TOF MS (DCTB + NaTFA): [CH_3_(C_5_H_9_NO)_2_OH+Na]^+^, *m/z* = 2036.39 observed.

**PEtOx_20_-COOH**

PEtOx-OH (18 g, 9.38 mmol, 1 eq.), DMAP (115 mg, 0.94 mmol, 0.1 eq.) and succinic anhydride (1.41 g, 14.1 mmol, 1.5 eq.) were dissolved in anhydrous 120 mL CH_2_Cl_2_. Triethylamine (1.42 g, 14.1 mmol, 1.5 eq.) was added to the mixture, which was stirred overnight at RT. The solvent was subsequently removed under reduced pressure, and the residue was dissolved in H_2_O and dialyzed against water (MWCO 0.1 to 0.5 kDa) for three days. Afterwards the solution was freeze dried. The product was obtained as a white powder (6.77 g, 38 %).

^1^H NMR (300 MHz, CDCl_3_): *δ* = 4.22 (br), 3.15 – 3.87 (br), 2.90 – 3.14 (m), 2.52 – 2.70 (m), 2.11 – 2.51 (m), 0.98 – 1.23 (br) ppm.

SEC (CHCl_3_/*i*-PrOH/NEt_3_, RI detection, PS calibration): M_n_ = 2410 g mol^-1^, *Đ* = 1.09.

MALDI-TOF MS (DCTB + NaTFA): [CH_3_(C_5_H_9_NO)_21_C_4_H_5_O_4_+Na]^+^, *m/z* = 2235.46, [CH_3_(C_5_H_9_NO)_21_C_4_H_4_O_4_+Na]^+^, *m/z* = 2257.45 observed.

***Figure S2:*** (A) ^1^H NMR spectra (CDCl_3_, 300 MHz) of PEtOx_20_-OAc (black), PEtOx_20_-OH (red) and PEtOx_20_-COOH (blue). (B) SEC elugrams (RID, CHCl_3_/*i*-PrOH/NEt_3_) of PEtOx_20_-OAc (black), PEtOx_20_-OH (red) and PEtOx_20_-COOH (blue). (C) Full MALDI-TOF mass spectra (DCTB, NaTFA) of PEtOx_20_-OAc (black), PEtOx_20_-OH (red) and PEtOx_20_-COOH (blue).


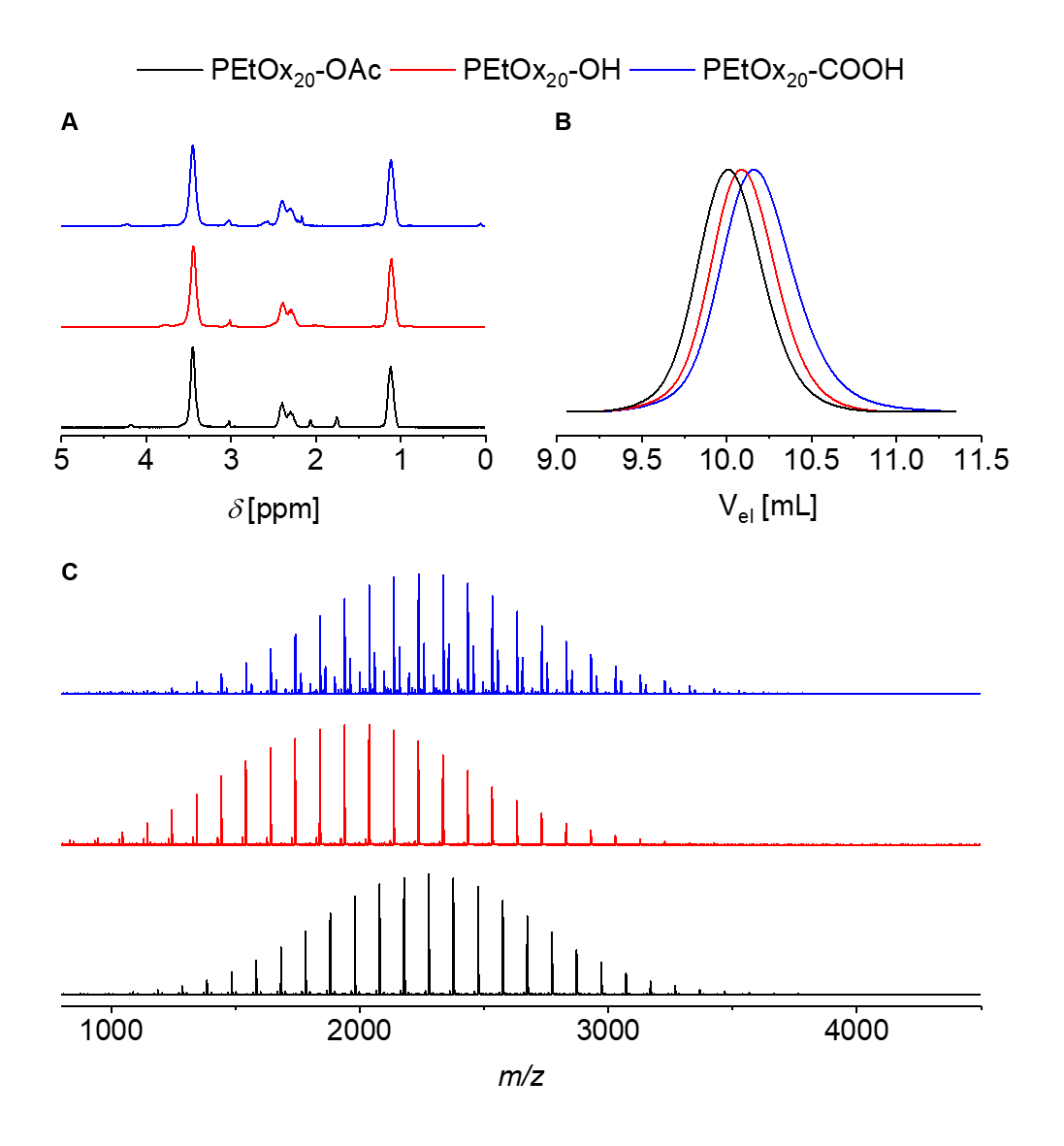


**PEtOx_20_-Lipid**

PEtOx-COOH (1 g, 0.47 mmol, 1 eq.), DMAP (5 mg, 0.047 mmol, 0.1 eq.), NHS (137 mg, 1.18 mmol, 2.5 eq.) and EDC (221 mg, 1.42 mmol, 3 eq.) were dissolved in 15 mL anhydrous CHCl_3_ and stirred for 3 h at RT. Ditetradecylamine (389 mg, 0.95 mmol, 2 eq.) was added and the mixture was stirred overnight at 45 °C. Subsequently, the mixture was washed with NaHCO_3_ and brine. The organic phase was dried over Na_2_SO_4_ and filtered. The solvent was removed under reduced pressure. The residue was dissolved in CH_2_Cl_2_ and precipitated in cold Et_2_O (−80 °C). The mixture was centrifuged and afterwards dried *in vacuo*. The product was obtained as a white powder (525 mg, 52 %).

^1^H NMR (300 MHz, CDCl_3_): *δ* = 4.19 (br), 3.46 (br), 3.14 – 3.31 (m), 2.92 – 3.08 (m), 2.62 (s), 2.17 – 2.52 (m), 1.39 – 1.63 (m), 1.25 (s), 1.12 (br), 0.87 (t) ppm.

SEC (CHCl_3_/*i*-PrOH/NEt_3_, RI detection, PS calibration): M_n_ = 3940 g mol^-1^, *Đ* = 1.05.

MALDI-TOF MS (DCTB + NaTFA): [CH_3_(C_5_H_9_NO)_19_C_4_H_4_O_3_NC_28_H_58_+Na]^+^, *m/z* = 2428.76 observed.


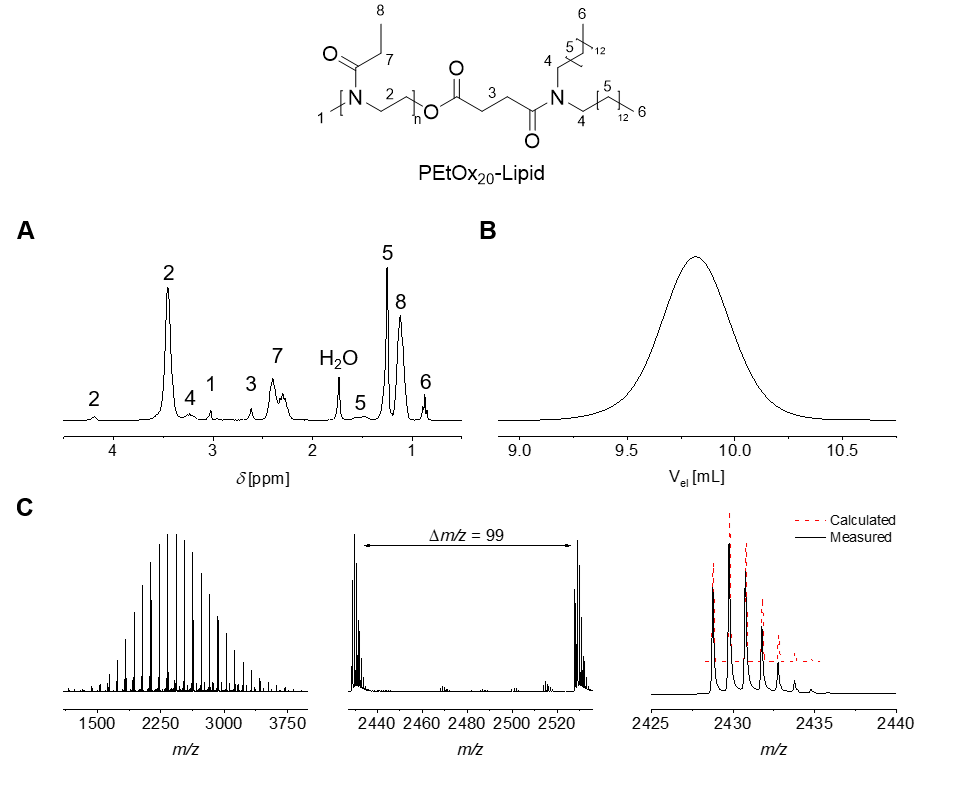


**Figure S3.** (A) ^1^H NMR spectrum (CDCl_3_, 300 MHz) of PEtOx_20_-Lipid and assignment of the signals to the schematic representation of its structure. (B) SEC elugram (RID, CHCl_3_/i-PrOH/NEt_3_) of PEtOx_20_-Lipid. (C) MALDI TOF mass spectrum of PEtOx_20_-Lipid (DCTB, NaTFA). From left to right: Full spectrum, display of the repeating unit of EtOx Δm/z = 99 and overlay of the isotopic pattern of the most abundant species (black: measured, red: calculated). The identified species was found as a sodium adduct [CH_3_(C_5_H_9_NO)_19_C_4_H_4_O_3_NC_28_H_58_+Na]^+^.

1. **Nanoparticle formulation:**

*Nanoparticle formulation trials*

Various materials and methods were tested on the mission to encapsulate pictilisib. These methods are briefly discussed below.

Nanoprecipitation method: 5 to 15 mg of polymer of choice (PLGA, DY635-PLGA, PLA-*b*-PEtOx, acetalated dextran (AcDex), or a 1:1 mixture of PEG-PLGA and PLGA) were dissolved in 1 mL acetone. Pictilisib was dissolved in DMSO at a concentration of 5 mg mL^-1^, and a specific volume of the stock solution was added to the organic polymeric phase to achieve a theoretical drug loading of 1.5 to 5 wt% of the polymer mass, depending on the formulation. The organic phase was then thoroughly vortexed and added dropwise using a syringe pump (Aladdin AL1000-220, World Precision Instruments, Friedberg, Germany) into 8 mL of a 0.3% (w/v) aqueous polyvinyl alcohol (PVA) solution at a flow rate of 2 mL min^−1^.

Single emulsion method: 15 mg of PLGA or AcDex were dissolved in 0.5 mL ethyl acetate and then 1 mL of a 3% (w/v) aqueous PVA solution was added on top. The resulting suspension was ultrasonicated for 10 sec and then poured into 4 mL of water. Ultrasonication was performed using a probe sonicator (Hielscher Sonotrode S26d2, Ø 2 mm, length ~120 mm) powered by an ultrasonic generator (UP200ST, Hielscher Ultrasonics, Teltow, Germany) at 100% cycle, 100 W power and 20% amplitude.

Hybrid lipid-polymer formulations: Basing on the protocol on work published previously by Ismail *et al*. (Ismail et al., 2024), 25 mg of PLGA was dissolved in 1 mL of acetonitrile and pictilisib was added at 5 wt% relative to the polymer. Lecithin and DSPE-PEG were dissolved in a 4 wt% ethanol aqueous solution and heated up to 65 °C. The organic (polymer and drug) solution was subsequently added to the aqueous lipid solution using a syringe pump dropwise at a flow rate of 2 mL min^−1^. Additionally, PVA was added afterwards at 25 wt% of the polymer mass.

All nanosuspensions were left to stir overnight at 800 rpm at RT to allow solvent evaporation when appropriate.

*Nanoparticle purification*

Depending on the size of the formulated nanoparticles, a suitable purification method was implemented. Nanoparticle formulations that were characterized by nanoparticle sizes below 150 nm were purified using ultrafiltration by transferring the NP dispersions into 15 mL Amicon filters (100,000 g mol^−1^ MWCO) and centrifuging at 4500 *g* for 30 min. The supernatant was then discarded, and the NPs were washed five times with Milli-Q water, before resuspending the sample in 1 mL of water. Bigger nanoparticles were purified by centrifuging the samples using a swing-bucket rotor at a fixed speed of 2880 *g* for 30 min at 20 °C (Centrifuge 5804 R, Eppendorf, Wesseling-Berzdorf, Germany). The supernatant was then discarded, the sample washed with Milli-Q water five times and then resuspended in 1 mL of water.

After checking the suspensions under a light microscope, all formulations revealed macroscopic drug precipitates. As such, separating the precipitates from the nanoparticles was essential to determine true encapsulation efficiency. So, the suspensions were passed through a 0.45 µm cellulose acetate (CA) filters, previously prewetted with Milli-Q water, and the filtrates were collected for further characterization.

The characterization of the resulting formulations is summarized in Table S1.

| **#** | **Method** | **Material composition** | **Mass [mg]** | **Solvent** | **Surfactant** | **Cargo (wt% polymer)** | **d_h_ (nm)** | **PDI** | **Drug precipitates before filtration?** | **EE (%) after filtration** | **LC (%) after filtration** |
| --- | --- | --- | --- | --- | --- | --- | --- | --- | --- | --- | --- |
| 1 | Nanoprecipitation | PLGA | 10 | Acetone | PVA (0.3%) | 5% | 185-207 | 0.05-0.18 | Yes | 4.64 | 0.23 |
| 2 |  | PLGA | 5 | Acetone | PVA (0.3%) | 5% | 188 | 0.08 | Yes | 2.69 | 0.27 |
| 3 |  | DY635-PLGA | 10 | Acetone | PVA (0.3%) | 5% | 178-194 | 0.04-0.2 | Yes | 7.7 | 0.38 |
| 4 |  | PLGA | 10 | Acetone | PVA (0.3%) | 1.5% | 235 | 0.04 | Yes | 15.89 | 0.24 |
| 5 |  | PLGA | 15 | Acetone | PVA (0.3%) | 3.3% | 258 | 0.05 | Yes | 12.78 | 0.43 |
| 6 |  | AcDex | 10 | Acetone | PVA (0.3%) | 5% | 176 | 0.14 | Yes | 3.75 | 0.19 |
| 7 |  | AcDex | 10 | Acetone | PVA (0.3%) | 1.5% | 170 | 0.11 | Yes | 12 | 0.18 |
| 8 |  | PLGA | 15 | Acetone | PVA (0.3%) | 5% | 224 | 0.2 | Yes | 2.61 | 0.13 |
| 9 |  | PLGA | 15 | Acetone | PVA (0.3%) | 1.5% | 211 | 0.15 | Yes | 5.5 | 0.083 |
| 10 |  | PLGA | 15 | Acetone | PVA (0.3%) | 5% | 208 | 0.1 | Yes | 0.73 | 0.04 |
| 11 |  | PLGA | 15 | Acetone | PVA (0.3%) | 1.5% | 182 | 0.05 | Yes | 8.13 | 0.12 |
| 12 |  | AcDex | 15 | Acetone | PVA (0.3%) | 5% | 164 | 0.25 | Yes | 8.32 | 0.416 |
| 13 |  | AcDex | 15 | Acetone | PVA (0.3%) | 1.5% | 167 | 0.08 | Yes | 17.74 | 0.27 |
| 14 |  | PEG-PLGA:PLGA (1:1) | 10 | Acetone | PVA (0.3%) | 5.0% | 123 | 0.13 | Yes | - | - |
| 15 |  | PLA-*b*-PEtOx | 10 | Acetonitrile | PVA (0.3%) | 5.0% | 119 | 0.12 | Yes | 10.4 | 0.52 |
| 16 | Single emulsion | PLGA | 15 | Ethyl acetate | PVA (1 mL, 3%) | 1.5% | 126 | 0.12 | Yes | 12.5 | 0.187 |
| 17 |  | AcDex | 15 | Ethyl acetate | PVA (1 mL, 3%) | 1.5% | 109 | 0.12 | Yes | 17.5 | 0.26 |
| 18 | Nanoprecipitation | Hybrid NPs: PLGA, DSPE-PEG, lecithin | 10:1:0.5 ratio | Acetonitrile | PVA (0.3%) | 5% | 116 | 0.17 | Yes | 11.7 | 0.51 |

***Table S1.*** Brief summary of example formulations that were unsuccessful in pictilisib encapsulation and their parameters (d_h_ = hydrodynamic diameter, PDI = polydispersity index, LC = loading capacity, EE = encapsulation efficiency).


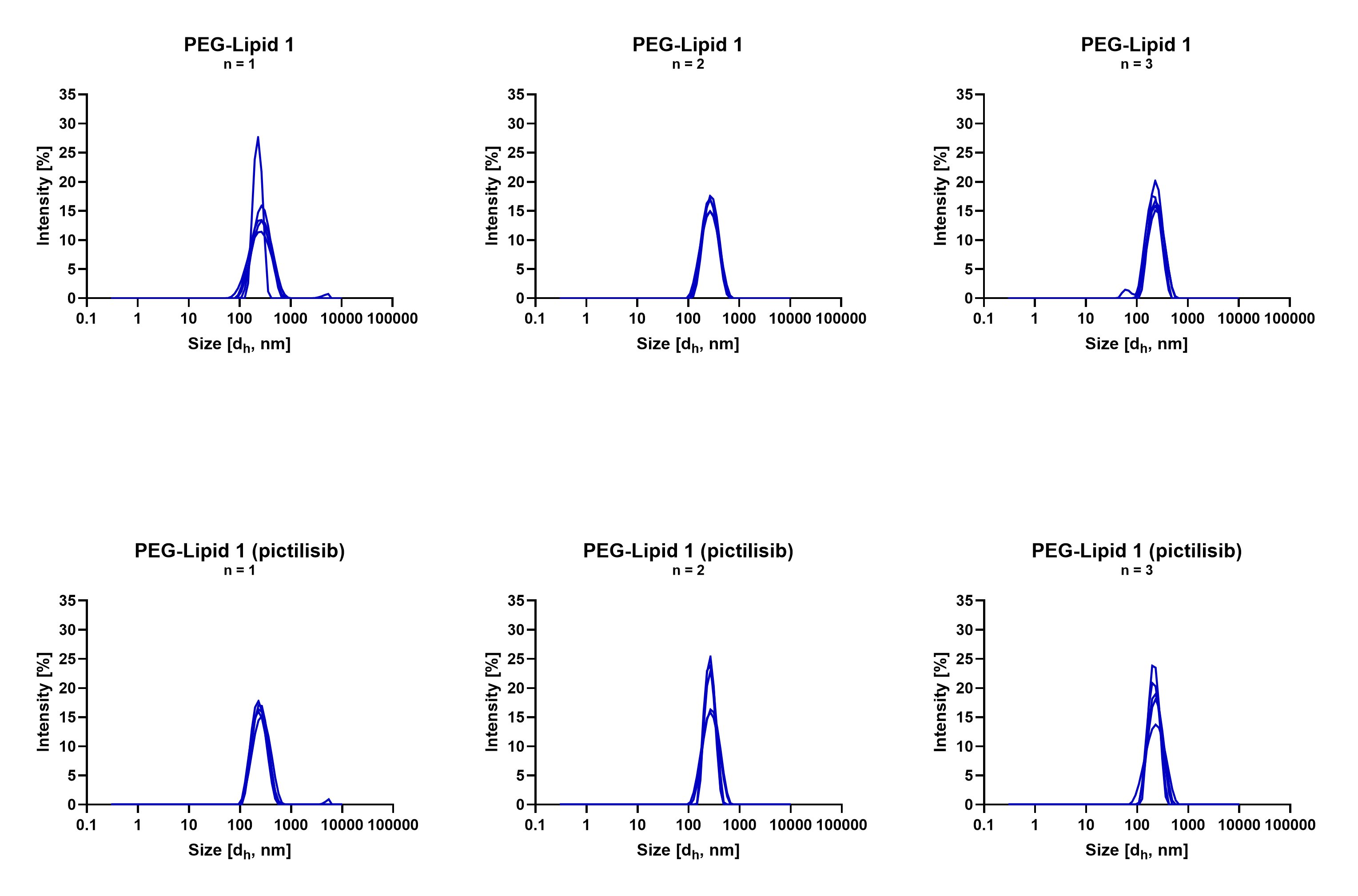

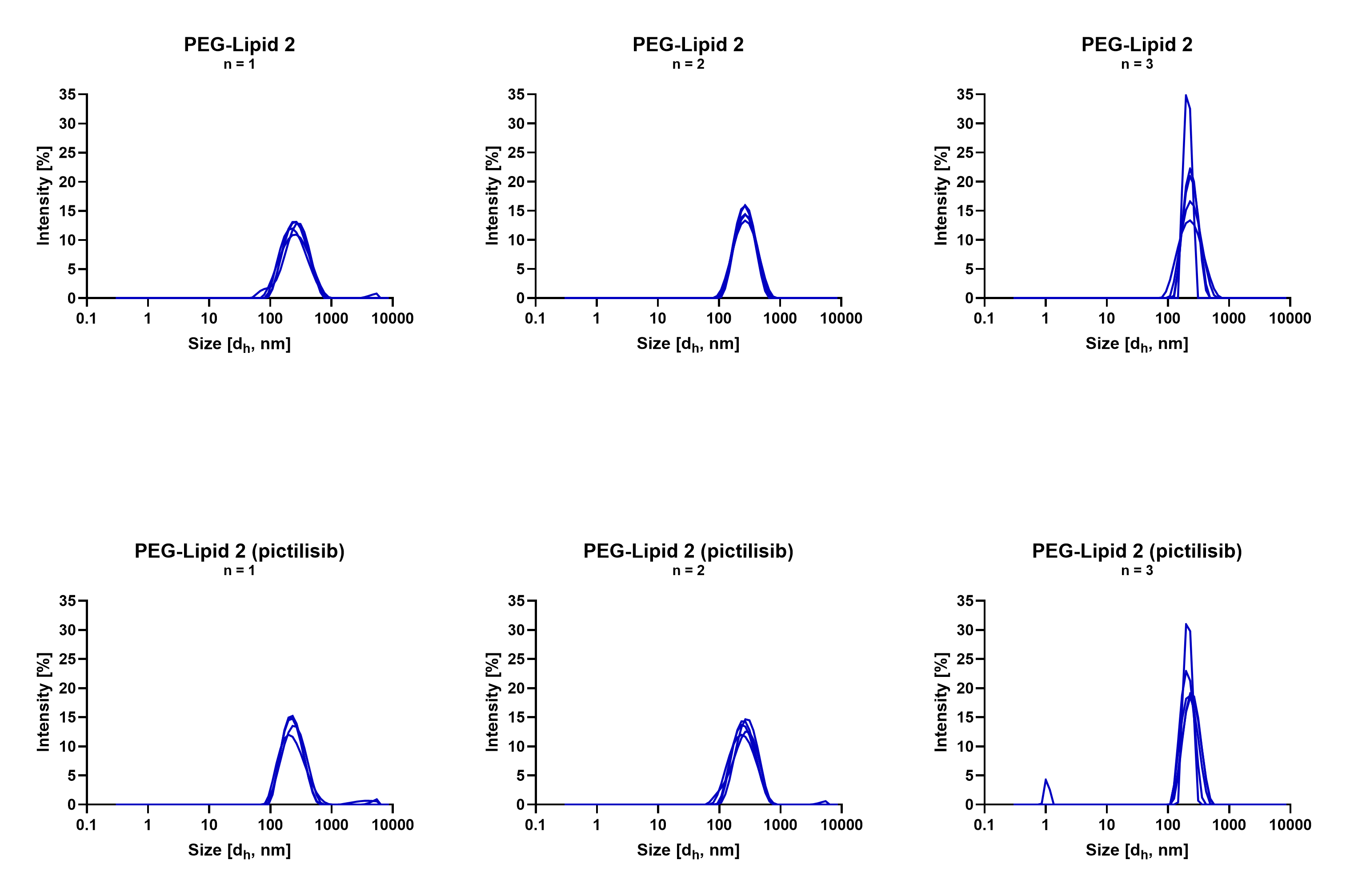


***Figure S4.*** *Continued*


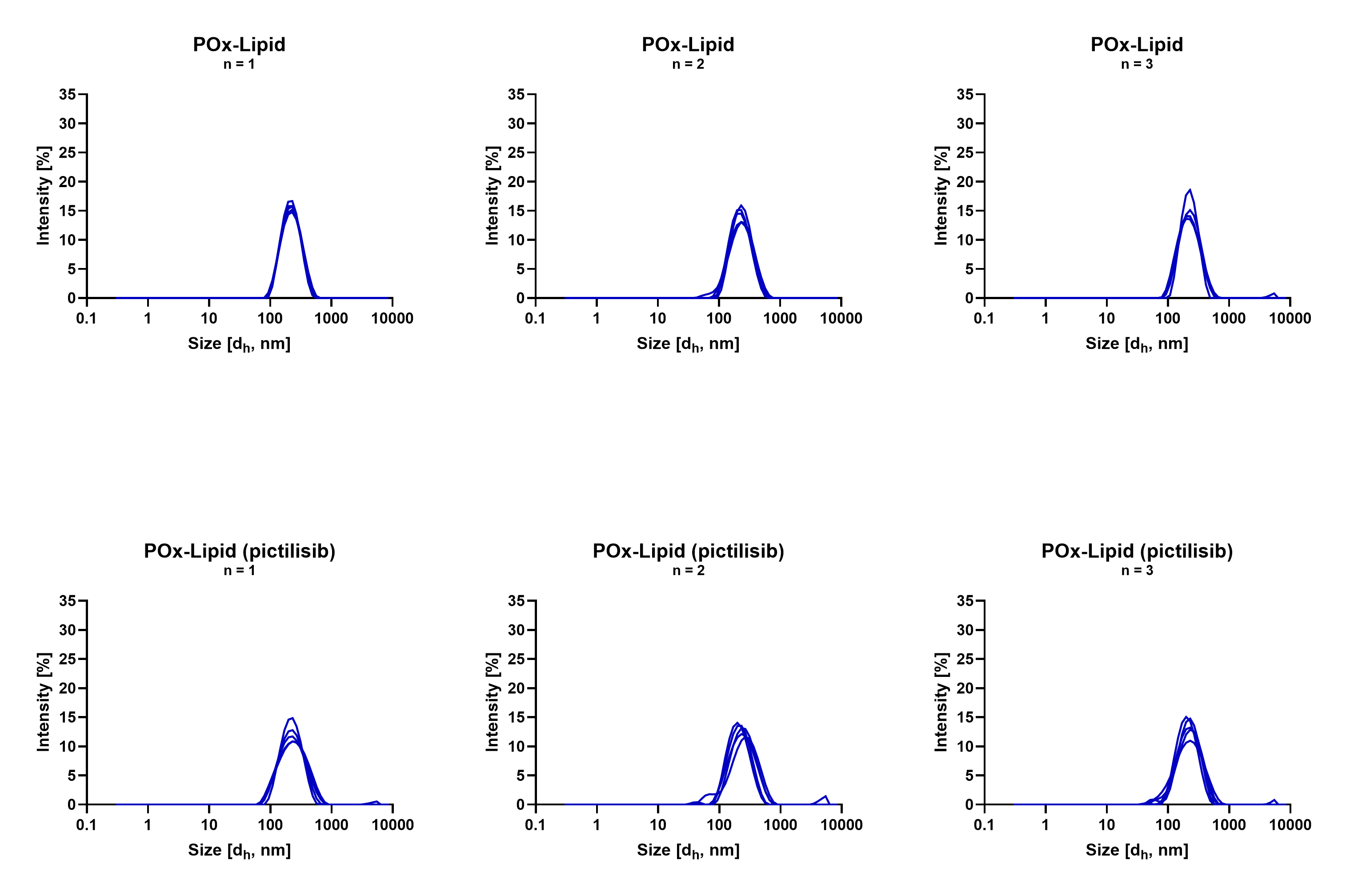


***Figure S4.*** Size distribution by intensity of all LNP formulations. Samples were diluted 1:100 in Milli-Q water and then analyzed by dynamic light scattering (DLS) using a 633 nm laser wavelength and a back-scattering angle of 174.7°. Each formulation was measured in five replicates. All formulations were prepared in triplicates (n=3), and all replicates are demonstrated.

***Table S2****.* Characteristics of the DiI-loaded LNPs (n = 1 technical replicate, n = 5 for DLS measurement).

| Composition | Nomenclature | Cargo | d_h_ (nm) | PDI | ζ (mV) |
| --- | --- | --- | --- | --- | --- |
| DMG-PEG2k, lecithin | PEG-Lipid 1 (DiI) | DiI [0.25 wt%] | 246 | 0.15 | +24 |
| PEtOx_20_-Lipid, lecithin | POx-Lipid (DiI) | DiI [0.25 wt%] | 201 | 0.12 | +21 |

1. **Biological characterization**

***Figure S5.*** *Continued*


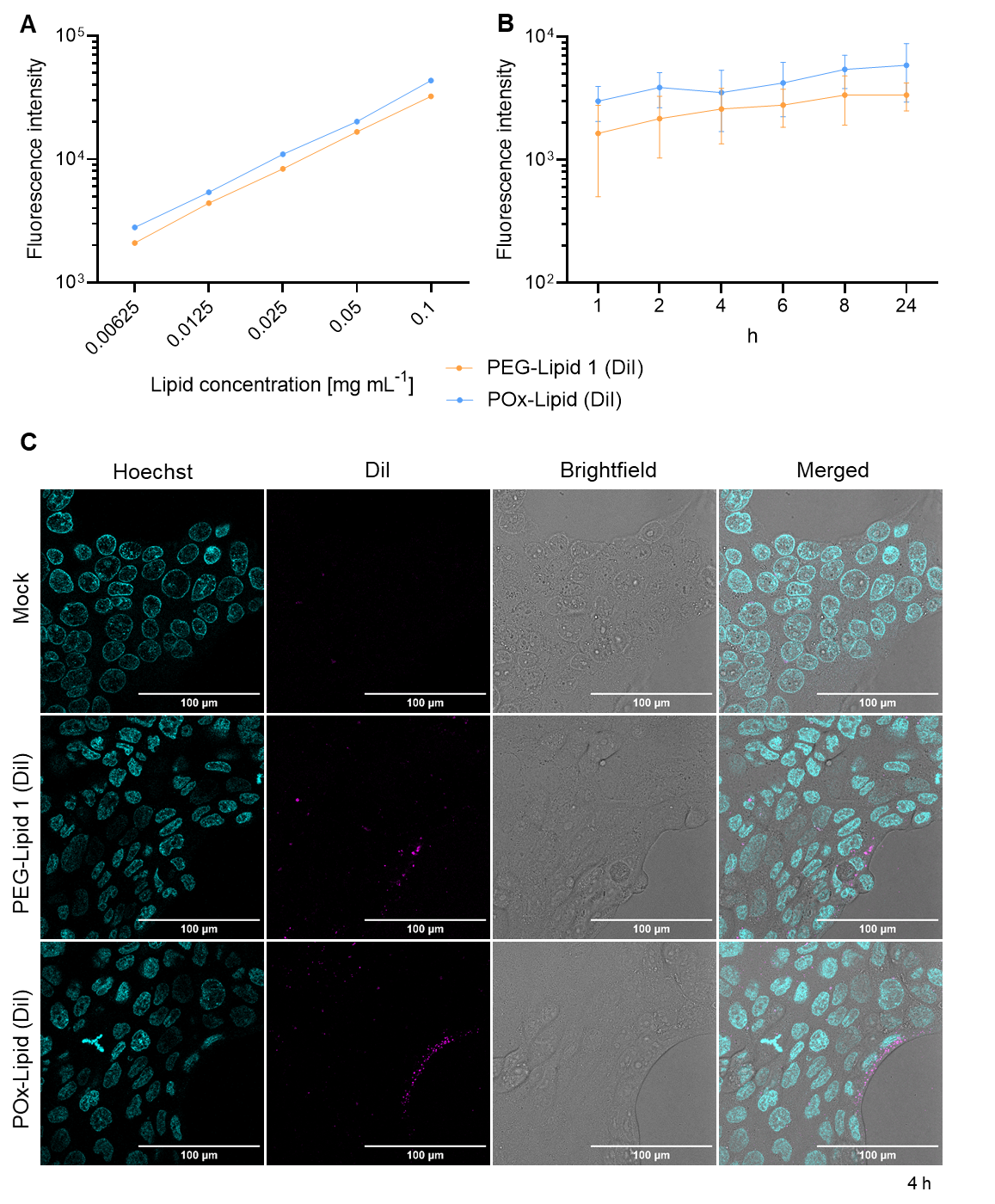

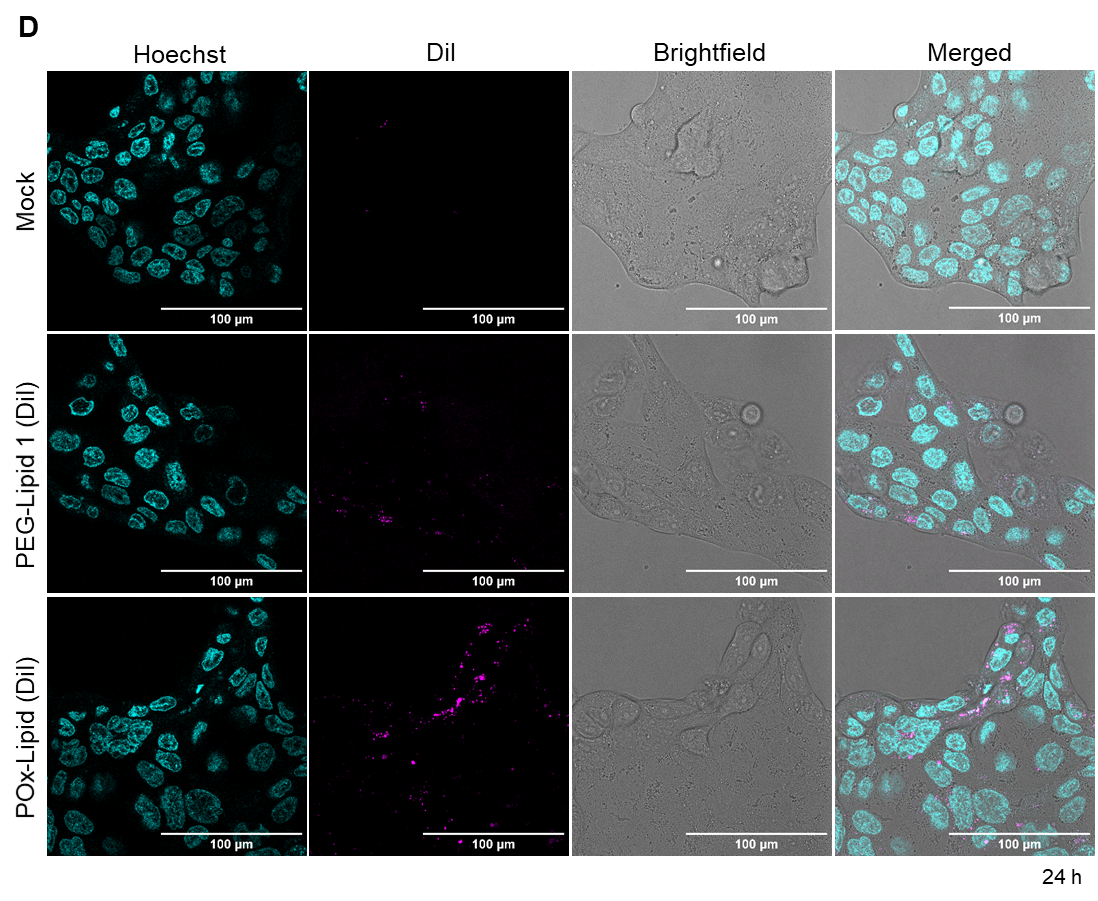


**Figure S5.** DiI-loaded LNPs were successfully internalized by Calu-3 cells. (A) The fluorescence of different dilutions of PEG-Lipid 1 (DiI) and POx-Lipid was measured using a TECAN Infinite 200 with an excitation at 540 nm and an emission at 610 nm. (B-D) Calu-3 cells were incubated with 0.1 mg mL^-1^ PEG-Lipid 1 (DiI), POx-Lipid (DiI) or exclusively with medium (mock) for a period of 1 to 24 h (B), or only for 4 h (C) or 24 h (D). The fluorescence of PEG-Lipid 1 (DiI) and POx-Lipid was measured using a TECAN Infinite 200 with an excitation at 540 nm and an emission at 610 nm (B). Nuclei were stained with Hoechst (cyan), LNPs are visualized through DiI fluorescence (magenta) and Calu-3 cells are visible through brightfield imaging. Scale bars represent 100 µm (C-D). Presented data is the mean of three independent experiments with three technical replicates (A, B) or a representation of three independent experiments (C, D).


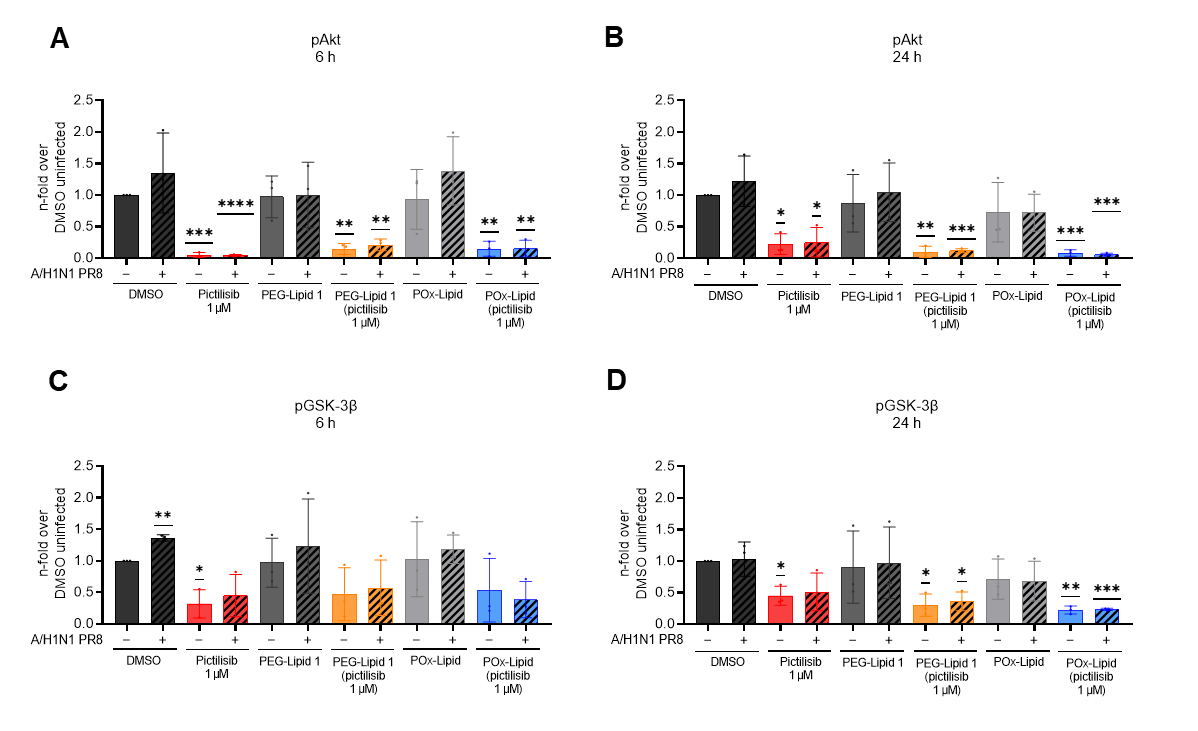


**Figure S6.** Quantification of the Western blot images from Figure 5G, H. Treatment with pictilisib and pictilisib-loaded LNPs regulates IAV-induced PI3K signaling. Calu-3 cells were pretreated for 1 h with 1 µM of the indicated compounds (pictilisib, PEG-Lipid 1 (pictilisib), POx-Lipid (pictilisib) or corresponding volumes of controls (DMSO, PEG-Lipid 1, POx-Lipid) and subsequently infected for 30 min with A/H1N1 PR8 (A, C: 3 MOI; B, D: 0.5 MOI). After removal of virus dilution, samples were treated again with the substances or controls for 6 h (A, C) or 24 h (B, D). Cell lysates were taken after the time periods for Western blot analysis. PI3K-mediated signaling was indirect detected via phosphorylation-levels of Akt (A, B) and GSK-3β (C, D). Data represent the mean ± SD of three independent experiments. The densitometry is represented as the mean fold protein expression of three independent experiments relative to DMSO-treated uninfected samples, normalized to vinculin and set to 1 (Fiji ImageJ 1.54i quantification). Statistical significance was determined by one-sample t-test comparing to a hypothetical mean of 1. *p < 0.05, **p < 0.01, ***p < 0.001, ****p < 0.0001. Comparisons without asterisks did not reach statistical significance.


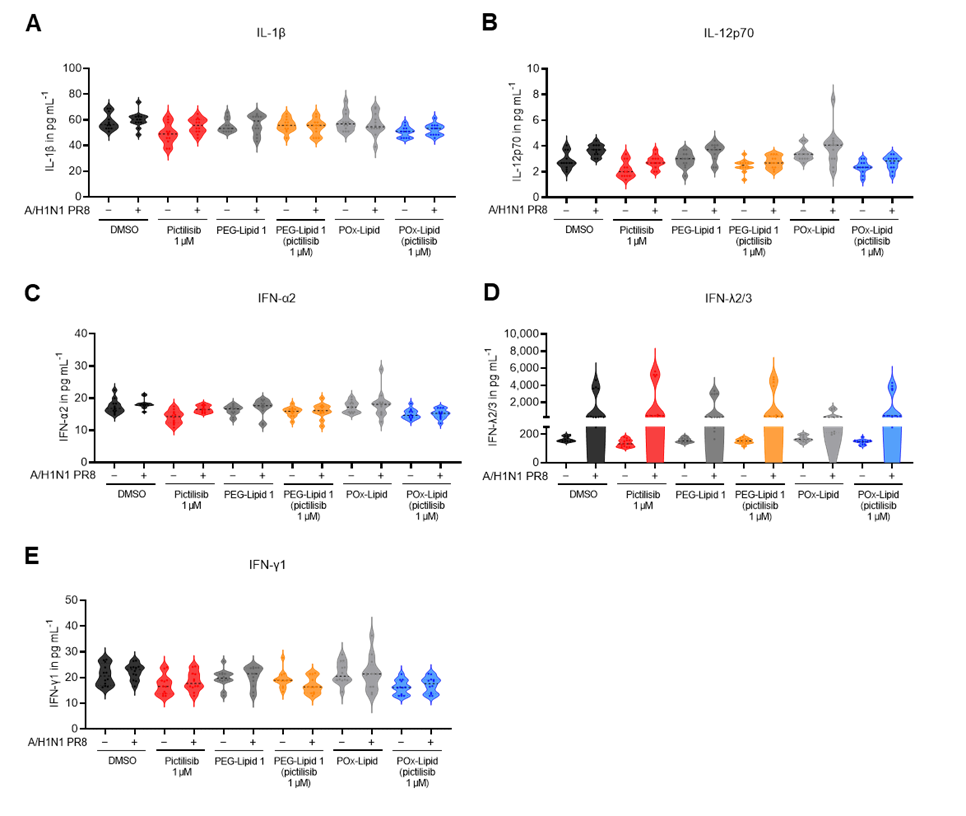


**Figure S7.** Additional cytokines measured as part of the LEGENDplex panel (see Figure 6). Treatment with pictilisib and pictilisib-loaded LNPs does not affect the IAV-induced cytokine production at 24 h p.i. in vitro. Calu-3 cells were pretreated with 1 µM pictilisib, PEG-Lipid 1 (pictilisib) or POx-Lipid (pictilisib) or corresponding volumes of controls for 1 h prior to infection and were then infected with 0.5 MOI of A/H1N1 PR8 for 30 min. Virus solution was removed after that, substances as well as respective controls were added again, and cells were incubated for further 23.5 h. Subsequently, supernatants were collected, and the amount of the indicated cytokines was detected using flow cytometry (LEGENDplex). Diagrams show three independent experiments with technical duplicates.


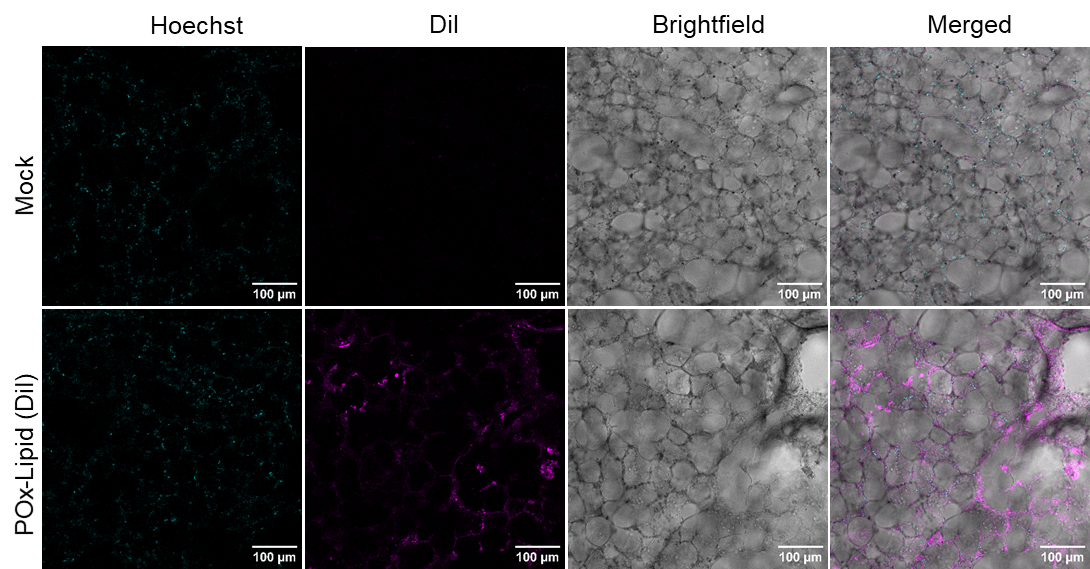


**Figure S8.** POx-Lipid (DiI) was successfully internalized into the cells of an ex vivo mouse lung slice. Ex vivo mouse lung slices were incubated with 0.1 mg mL^-1^ POx-Lipid (DiI) or only medium (mock) for 24 h. Nuclei were stained with Hoechst (cyan). The POx-Lipid is visualized through DiI fluorescence (magenta). Lung structure is shown through brightfield imaging. Scale bars represent 100 µm. The shown images are a representation of three independent mice.


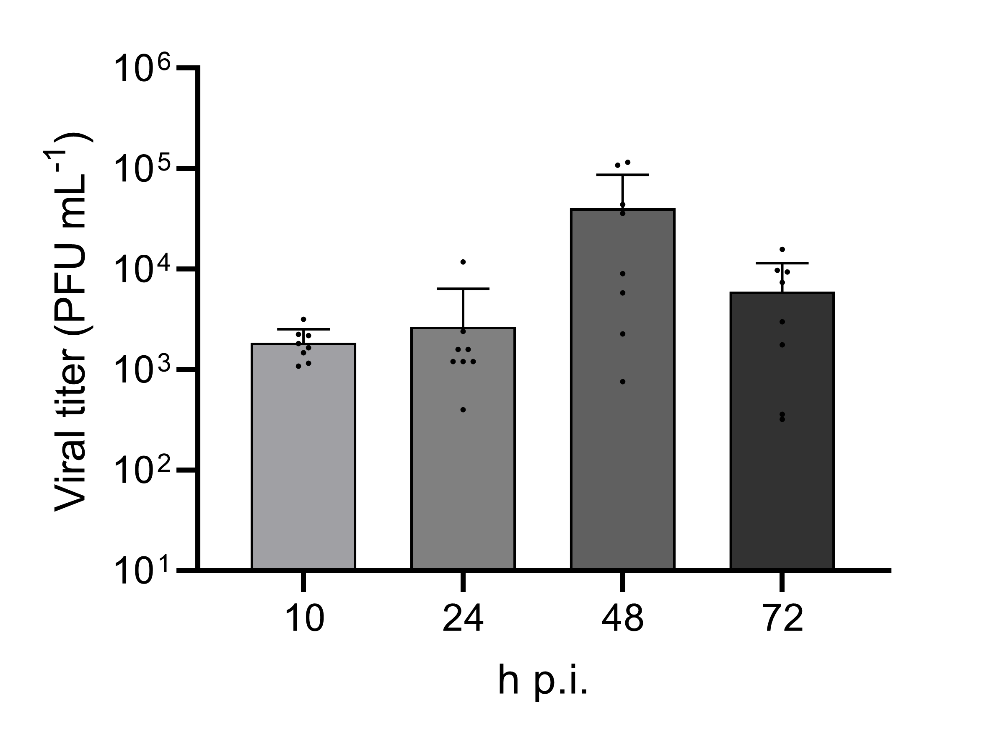


**Figure S9.** Kinetics of influenza virus A/H1N1pdm09 replication in ex vivo mouse lung slices. Mouse lung was extracted, cut into slices, infected with A/H1N1pdm09 Jena5258 for 3 h. Subsequently, the medium was replaced and the tissue was further incubated. At 10, 24, 48 and 72 h p.i. supernatants were used to determine progeny virus titers (PFU mL^-1^) by plaque assay. The mean + SD of four independent mice with two technical replicates is depicted.


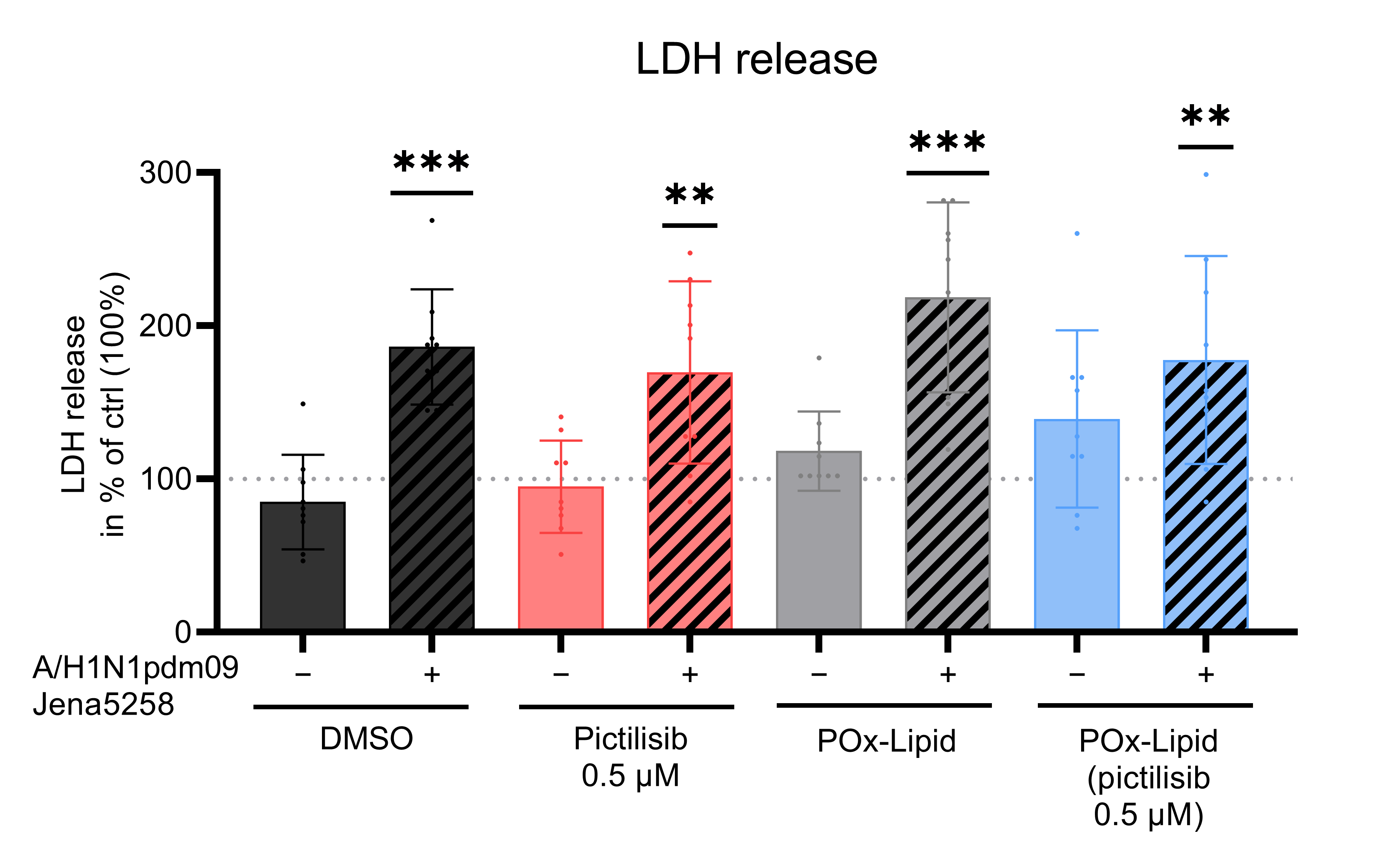


***Figure S10.*** IAV infection has an impact on LDH release of the lung slices. Ex vivo mouse lung slices were infected for 3 h with A/H1N1pdm09 Jena5258 or left untreated and treated until 48 h p.i. with 0.5 µM pictilisib, POx-Lipid (pictilisib) or respective volumes of DMSO and POx-Lipid. Supernatants were used for LDH measurement. Only medium-treated samples served as spontaneous LDH release control (ctrl) and were set to 100%. The data represent the mean ± SD of three independent mice with three technical replicates. Statistical significance was determined by one sample t-test with the hypothetical mean set to 100. ***p* < 0.01, ****p* < 0.001. Comparisons without asterisks did not reach statistical significance.


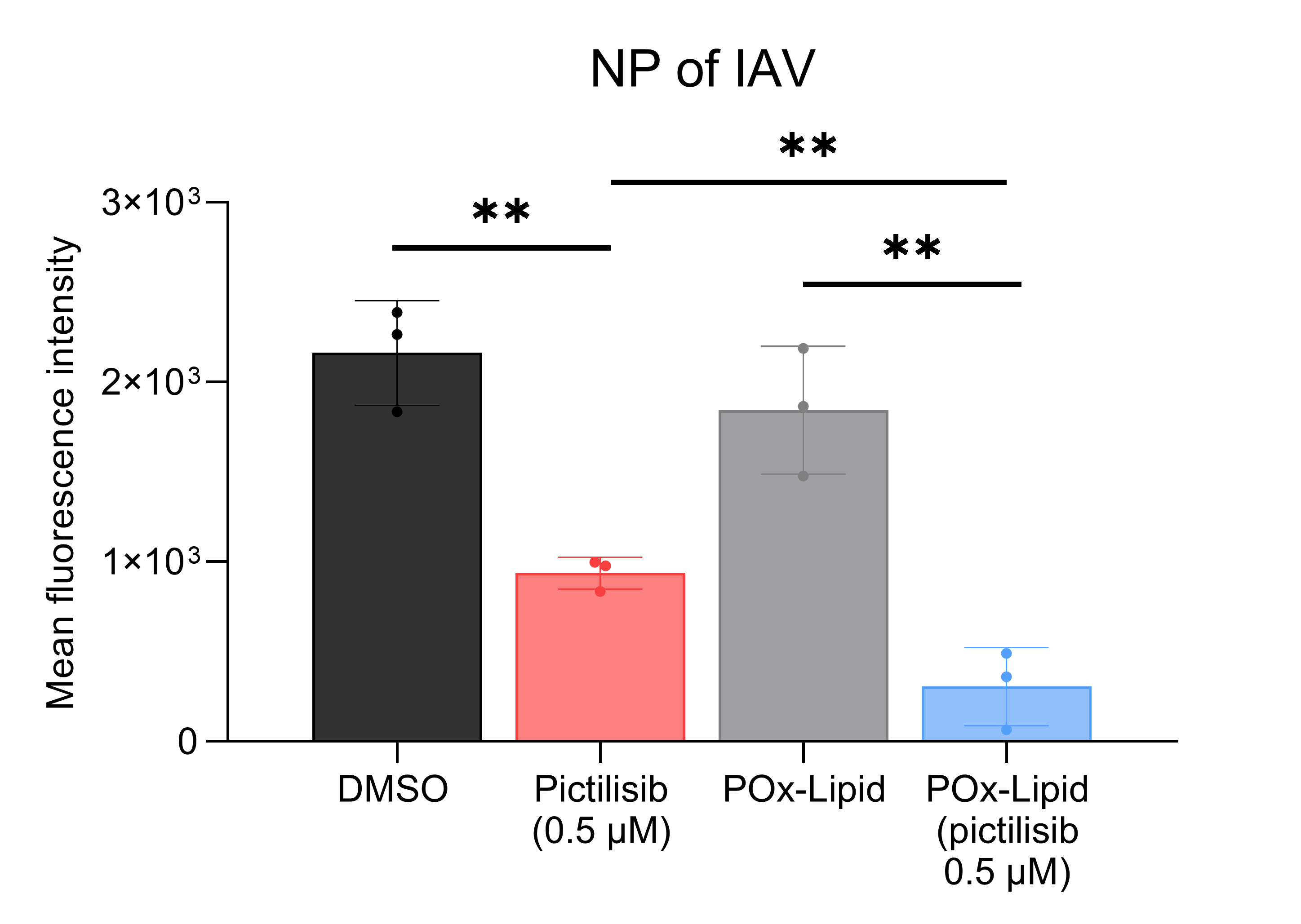


**Figure S11.** Quantification of the viral NP signal from Figure 7C and 7D. In the presence of pictilisib and POx-Lipid (pictilisib) IAV replication is reduced in ex vivo mouse lung slices. Ex vivo mouse lung slices were infected for 3 h with A/H1N1pdm09 Jena5258 or left uninfected and treated until 48 h p.i. with 0.5 µM pictilisib, POx-Lipid (pictilisib) or respective volumes of DMSO and POx-Lipid. The viral NP fluorescence signals were quantified based on mean fluorescence intensities per image. The mean ± SD of three independent mice with three images per mouse were analyzed. To account for background signal, the overall mean fluorescence intensity of all analyzed images from uninfected samples within each treatment group was subtracted from the corresponding values of infected samples. Statistical significance was analyzed by unpaired two-tailed t-tests. **p < 0.01. Comparisons without asterisks did not reach statistical significance.


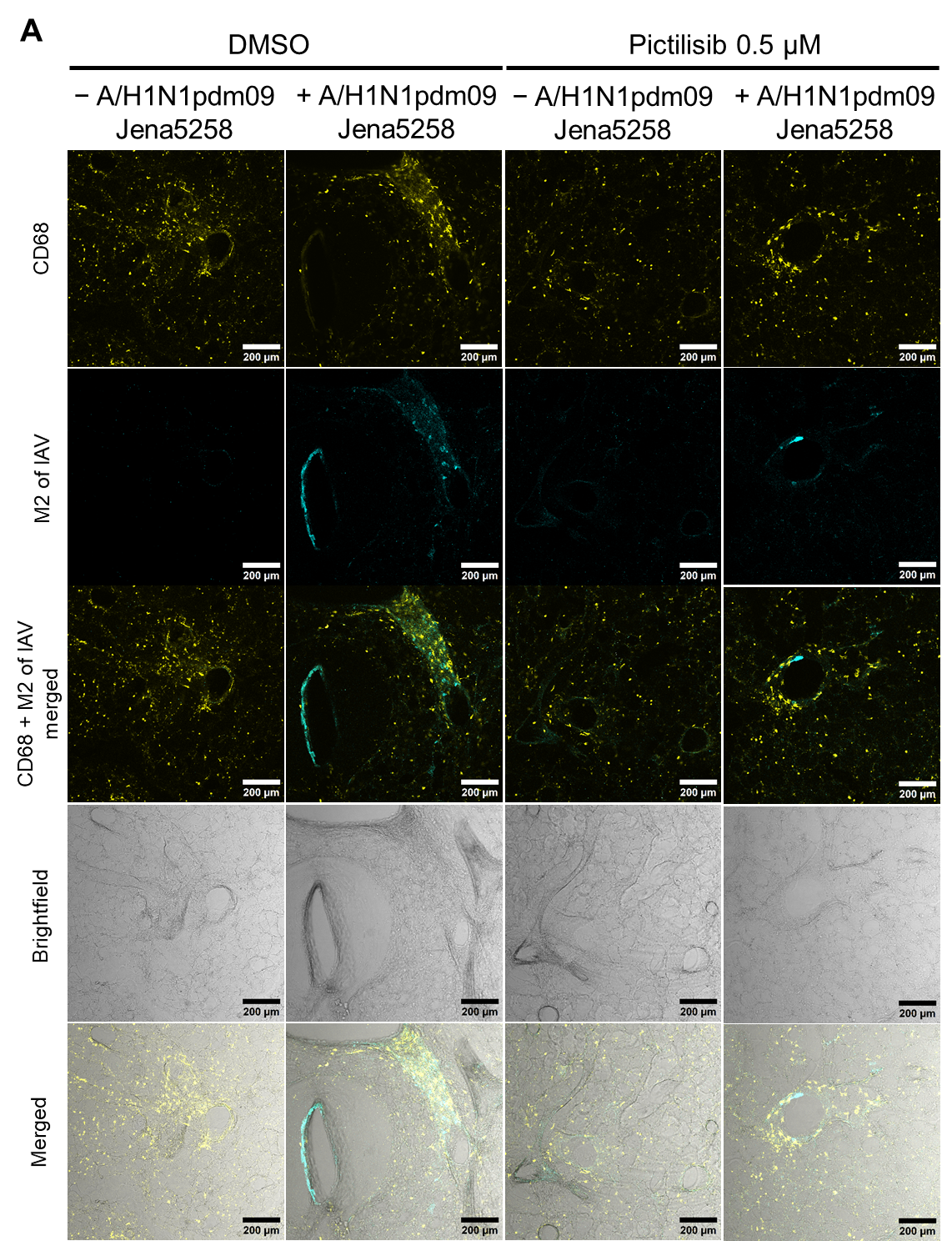


***Figure S12.*** *Continued*


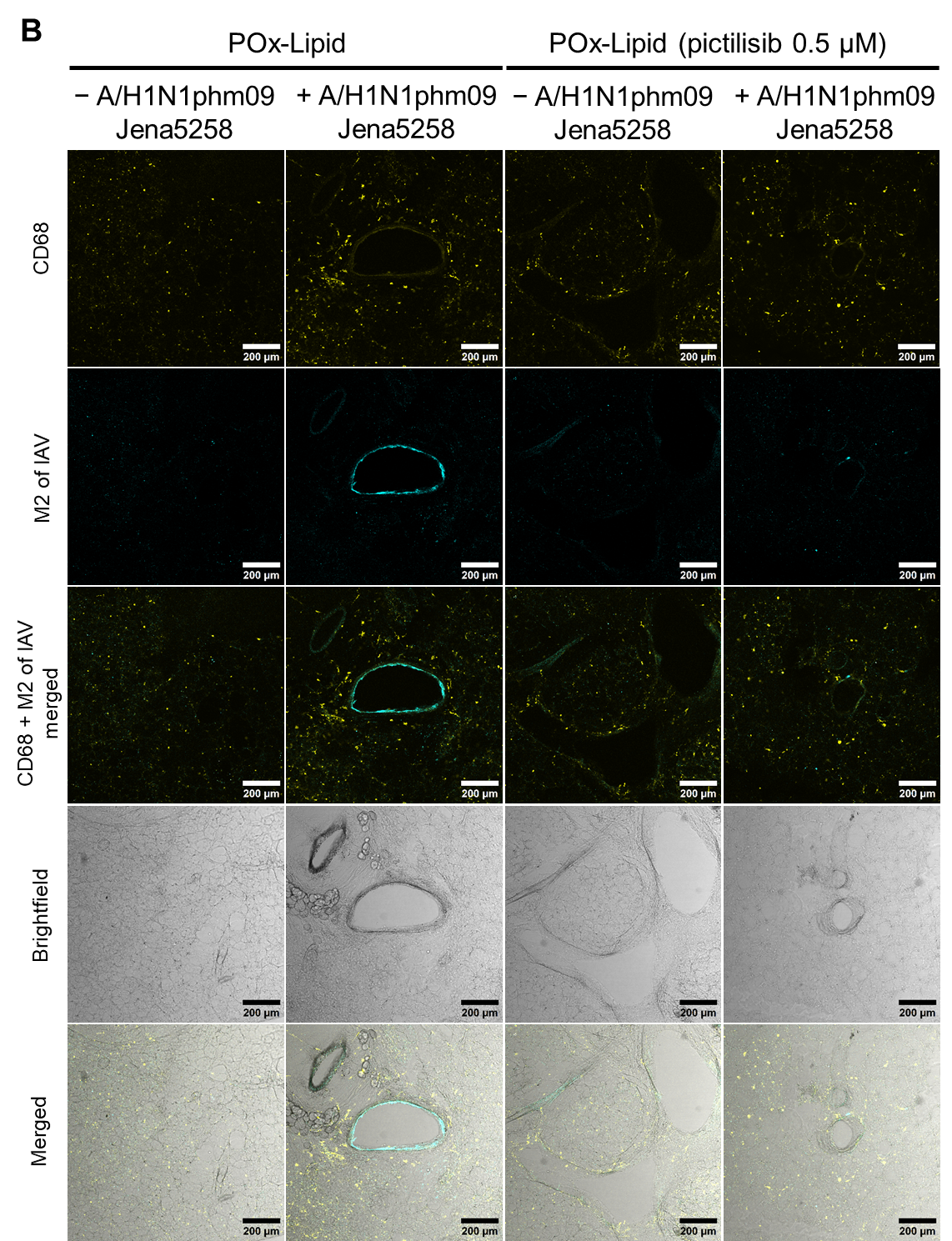


***Figure S12.*** *Continued*


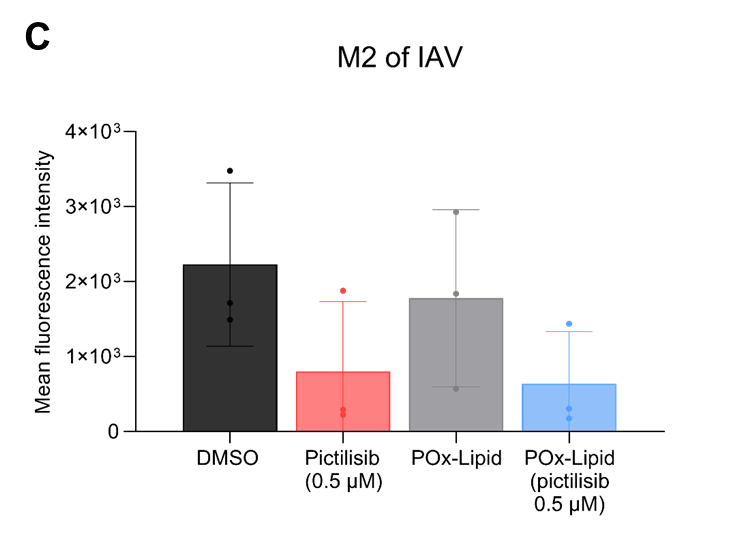


***Figure S12.*** In presence of pictilisib and POx-Lipid (pictilisib) IAV replication is reduced in *ex vivo* mouse lung slices. *Ex vivo* mouse lung slices were infected with A/H1N1pdm09 Jena5258 for 3 h or left uninfected and afterwards treated with 0.5 µM pictilisib, POx-Lipid (pictilisib) or respective volumes of the controls (DMSO, POx-Lipid) until 48 h p.i. (A, B) Alveolar macrophages were stained with a rat anti-CD68 primary antibody and an anti-rat Cy3 secondary antibody (yellow). Viral matrix protein 2 (M2) is stained with a rabbit anti-IAV M2 primary- and an anti-rabbit AF488 secondary antibody (cyan). Lung structure is shown through brightfield imaging. Scale bars represent 200 µm. Depicted is one out of three independent mice. (C) The viral M2 fluorescence signals were quantified based on mean fluorescence intensities per image. The mean ± SD of three independent mice with three images per mouse were analyzed. To account for background signal, the overall mean fluorescence intensity of all analyzed images from uninfected samples within each treatment group was subtracted from the corresponding values of infected samples. Statistical significance was analyzed by unpaired two-tailed t-tests. Comparisons without asterisks did not reach statistical significance.

**References**

Ismail, J., Klepsch, L. C., Dahlke, P., Tsarenko, E., Vollrath, A., Pretzel, D., Jordan, P. M., Rezaei, K., Czaplewska, J. A., Stumpf, S., Beringer-Siemers, B., Nischang, I., Hoeppener, S., Werz, O., & Schubert, U. S. (2024). PEG-Lipid-PLGA Hybrid Particles for Targeted Delivery of Anti-Inflammatory Drugs. *Pharmaceutics*, *16*(2).

Kauffman, K. J., Do, C., Sharma, S., Gallovic, M. D., Bachelder, E. M., & Ainslie, K. M. (2012). Synthesis and characterization of acetalated dextran polymer and microparticles with ethanol as a degradation product. *ACS Appl Mater Interfaces*, *4*(8), 4149-4155.

Press, A. T., Traeger, A., Pietsch, C., Mosig, A., Wagner, M., Clemens, M. G., Jbeily, N., Koch, N., Gottschaldt, M., Beziere, N., Ermolayev, V., Ntziachristos, V., Popp, J., Kessels, M. M., Qualmann, B., Schubert, U. S., & Bauer, M. (2014). Cell type-specific delivery of short interfering RNAs by dye-functionalised theranostic nanoparticles. *Nat Commun*, *5*, 5565.

Stafast, M. L. S., M.; Poudel, P.; Engel, N.; Yin, C.; Scheuer, K.; Weber, C.; Schacher H. F.; Jandt, K. D.; Schubert, U. S. (2024). POxylated stereocomplexes from PEtOx-b-PLA diblock copolymers. *European Polymer Journal*, *221*(113545).
